# Supplementary figures and images for: Conditional chemoconnectomics (cCCTomics) as a strategy for efficient and conditional targeting of chemical transmission
Source: eLife. 2024 Apr 30;12:RP91927. doi: 10.7554/eLife.91927 (PMC11060718; doi:10.7554/eLife.91927)

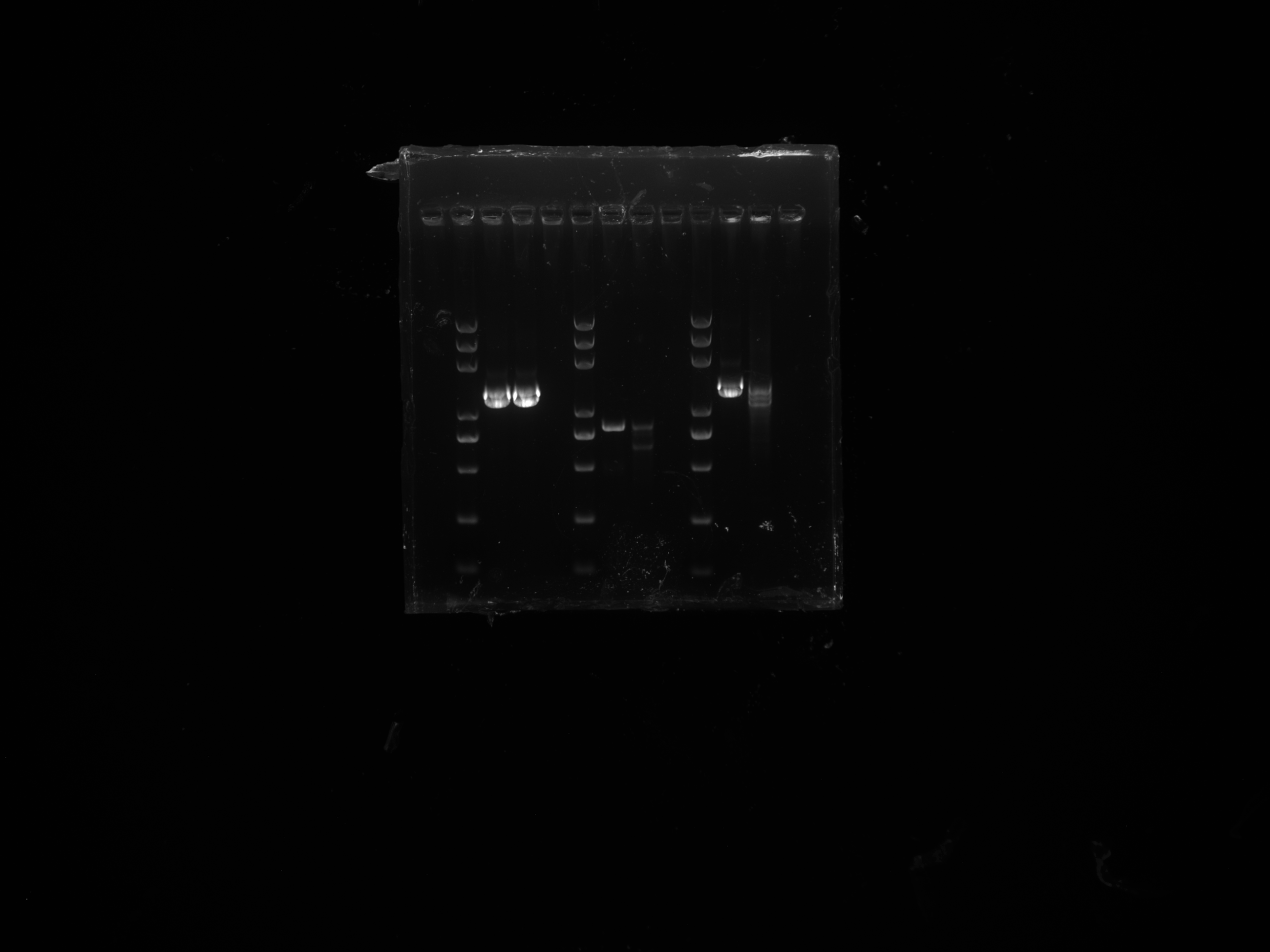

Supplement: Figure 2—figure supplement 1—source data 1. [file elife-91927-fig2-figsupp1-data1.zip › Figure 2-figure supplement 1-Source data.Tif]

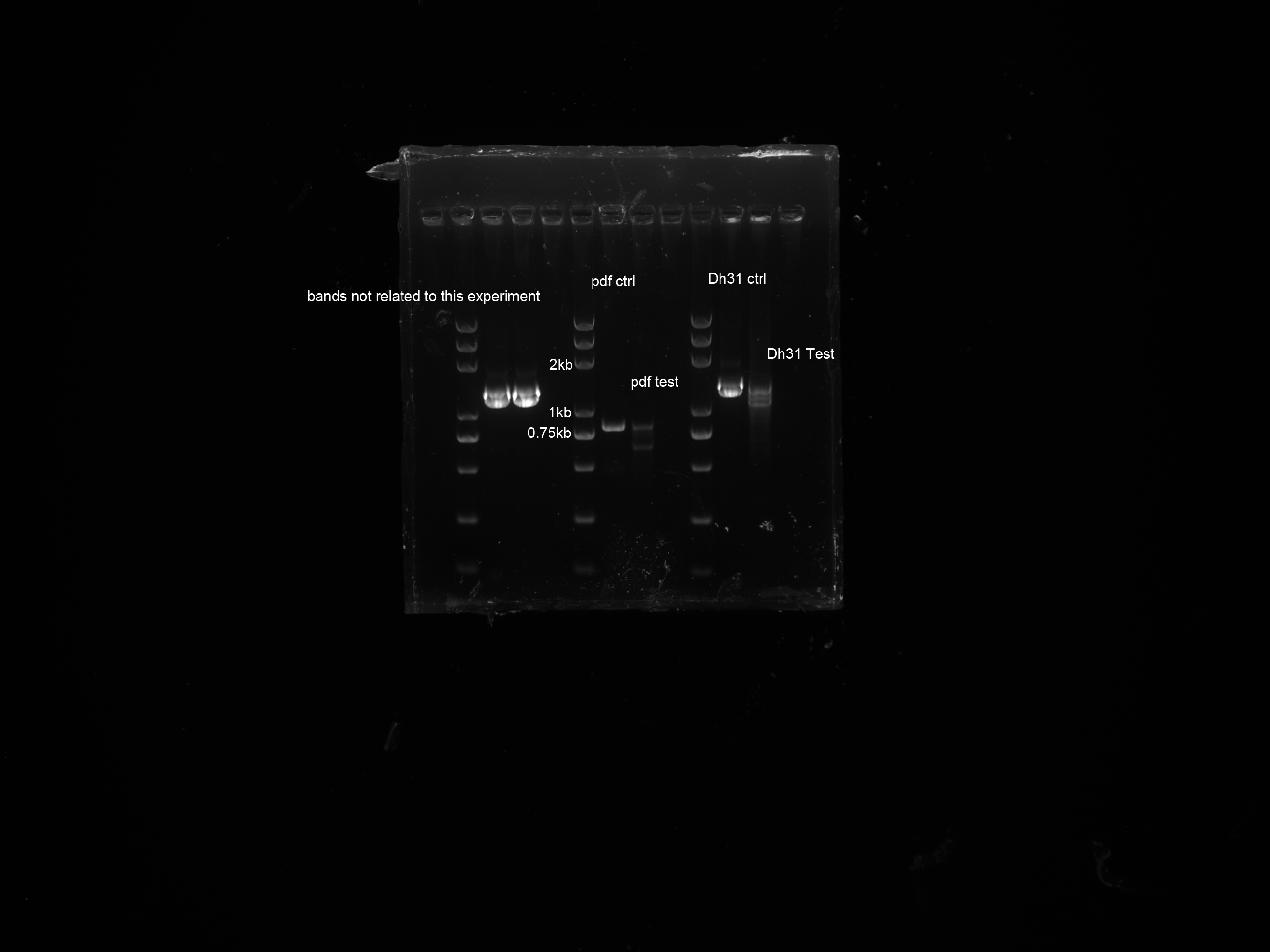

Supplement: Figure 2—figure supplement 1—source data 2. [file elife-91927-fig2-figsupp1-data2.zip › Figure 2-figure supplement 1-Source data2/Figure 2-figure supplement 1-Source data.Tif]
