# Supplementary material for: Conditional chemoconnectomics (cCCTomics) as a strategy for efficient and conditional targeting of chemical transmission
Source: eLife. 2024 Apr 30;12:RP91927. doi: 10.7554/eLife.91927 (PMC11060718; doi:10.7554/eLife.91927)
Supplement: Supplementary file 1. [file elife-91927-supp1.docx]

**List of Conditional chemoconectome knockin flies**

| **cCCT NO.** | **CG number** | **Gene Symbol** | **cCCT NO.** | **CG number** | **Gene Symbol** |
| --- | --- | --- | --- | --- | --- |
| CCT1002C | CG11325 | AkhR | CCT1087C | CG42244 | Octβ3R |
| CCT1003C | CG14375 | CCHa2 | CCT1088C | CG7411 | Ort |
| CCT1004C | CG14593 | CCHa2-R | CCT1089C | CG1543 | TβH |
| CCT1007C | CG6936 | mth | CCT1093C | CG13968 | sNPF |
| CCT1008C | CG31147 | mthl11 | CCT1097C | CG15520 | Capa |
| CCT1009C | CG17084 | mthl9 | CCT1098C | CG4910 | CCAP |
| CCT1010C | CG6496 | Pdf | CCT1099C | CG11318 | CG11318 |
| CCT1011C | CG8784 | PK2-R1 | CCT1102C | CG13229 | CG13229 |
| CCT1012C | CG13687 | Ptth | CCT1103C | CG15556 | CG15556 |
| CCT1013C | CG14734 | Tk | CCT1107C | CG30340 | CG30340 |
| CCT1014C | CG6515 | TkR86C | CCT1108C | CG32447 | CG32447 |
| CCT1016C | CG13633 | AstA | CCT1109C | CG33639 | CG33639 |
| CCT1019C | CG3302 | Crz | CCT1110C | CG33696 | CNMaR |
| CCT1020C | CG10698 | CrzR | CCT1111C | CG34411 | Lgr4 |
| CCT1021C | CG2902 | Nmdar1 | CCT1113C | CG43795 | CG43795 |
| CCT1024C | CG32540 | CCKLR-17D3 | CCT1114C | CG44153 | CG44153 |
| CCT1025C | CG33517 | D2R | CCT1117C | CG12345 | Cha |
| CCT1026C | CG13094 | Dh31 | CCT1118C | CG13936 | CNMamide |
| CCT1027C | CG32843 | Dh31-R | CCT1119C | CG8380 | DAT |
| CCT1028C | CG18090 | Dsk | CCT1120C | CG9887 | dVGLUT |
| CCT1029C | CG6440 | Ms | CCT1121C | CG5400 | Eh |
| CCT1030C | CG43745 | MsR2 | CCT1122C | CG18105 | ETH |
| CCT1034C | CG13480 | Lk | CCT1123C | CG5911 | ETHR |
| CCT1035C | CG10626 | Lkr | CCT1124C | CG2346 | FMRFa |
| CCT1036C | CG4128 | nAChR α6 | CCT1126C | CG15274 | GABA-B-R1 |
| CCT1037C | CG33976 | Octβ2R | CCT1128C | CG14994 | gad1 |
| CCT1039C | CG14358 | CCHa1 | CCT1129C | CG8442 | GluRIA |
| CCT1040C | CG30106 | CCHa1-R | CCT1130C | CG43743 | GluRIB |
| CCT1041C | CG13575 | CG13575 | CCT1131C | CG6992 | GluRIIA |
| CCT1042C | CG13995 | CG13995 | CCT1133C | CG4226 | GluRIIC |
| CCT1043C | CG33495 | Dup99B | CCT1134C | CG18039 | GluRIID |
| CCT1044C | CG31720 | mthl15 | CCT1135C | CG31201 | GluRIIE |
| CCT1047C | CG1147 | NPFr | CCT1136C | CG14723 | HisCl1 |
| CCT1050C | CG16752 | SPR | CCT1137C | CG13586 | ITP |
| CCT1051C | CG14871 | Trissin | CCT1138C | CG7665 | Lgr1 |
| CCT1052C | CG34381 | TrissinR | CCT1143C | CG8985 | MsR1 |
| CCT1053C | CG16720 | 5-HT1A | CCT1145C | CG32853 | mthl12 |
| CCT1055C | CG12073 | 5HT7 | CCT1147C | CG30018 | mthl13 |
| CCT1056C | CG14919 | AstC | CCT1148C | CG17795 | mthl2 |
| CCT1057C | CG14575 | CapaR | CCT1149C | CG6536 | mthl4 |
| CCT1058C | CG33344 | CCAP-R | CCT1150C | CG6965 | mthl5 |
| CCT1059C | CG13579 | CG13579 | CCT1151C | CG16992 | mthl6 |
| CCT1060C | CG31760 | CG31760 | CCT1152C | CG7476 | mthl7 |
| CCT1061C | CG32547 | CG32547 | CCT1153C | CG32475 | mthl8 |
| CCT1062C | CG18314 | DopEcR | CCT1156C | CG32538 | nAChR α7 |
| CCT1065C | CG3454 | HDC | CCT1158C | CG34388 | natalisin |
| CCT1066C | CG4395 | hec | CCT1159C | CG3441 | Nplp1 |
| CCT1068C | CG6456 | Mip | CCT1161C | CG15361 | Nplp4 |
| CCT1070C | CG6530 | mthl3 | CCT1164C | CG13565 | Orcokinin |
| CCT1071C | CG5610 | nAChR α1 | CCT1165C | CG15284 | Pburs |
| CCT1072C | CG6844 | nAChR α2 | CCT1166C | CG31660 | pog |
| CCT1073C | CG2302 | nAChR α3 | CCT1167C | CG7105 | Proc |
| CCT1074C | CG11348 | nAChRβ1 | CCT1168C | CG6986 | Proc-R |
| CCT1075C | CG6798 | nAChRβ2 | CCT1169C | CG10537 | Rdl |
| CCT1076C | CG6919 | oa2 | CCT1170C | CG8930 | rk |
| CCT1078C | CG7395 | sNPF-R | CCT1172C | CG5811 | RYa-R |
| CCT1079C | CG11895 | stan | CCT1174C | CG33527 | SIFa |
| CCT1080C | CG9122 | TRH | CCT1175C | CG10823 | SIFaR |
| CCT1081C | CG1056 | 5-HT2A | CCT1176C | CG3171 | Tre1 |
| CCT1082C | CG42796 | 5HT2B | CCT1177C | CG7431 | TyrR |
| CCT1083C | CG9753 | AdoR | CCT1179C | CG8394 | vGAT |
| CCT1084C | CG18208 | Octα2R | CCT1183C | CG5621 | Grik |
| CCT1085C | CG18741 | DopR2 | CCT1186C | CG8681 | clumsy |
| CCT1086C | CG32476 | mthl14 | CCT1189C | CG12344 | CG12344 |
