# Supplementary material for: Conditional chemoconnectomics (cCCTomics) as a strategy for efficient and conditional targeting of chemical transmission
Source: eLife. 2024 Apr 30;12:RP91927. doi: 10.7554/eLife.91927 (PMC11060718; doi:10.7554/eLife.91927)
Supplement: Supplementary file 2. [file elife-91927-supp2.docx]

**cCCT knockin strategy can’t rescue all knockout phenotype**

| **CG No.** | **Gene Symbol** | **attP KO phenotype** | **cCCT phenotype** |
| --- | --- | --- | --- |
| CG15520 | Capa | lethal | viable |
| CG12345 | ChAT | lethal | viable |
| CG5400 | Eh | lethal | viable |
| CG5911 | ETHR | lethal | viable |
| CG10537 | Rdl | lethal | viable |
| CG14575 | CapaR | lethal | lethal |
| CG14994 | gad1 | lethal | lethal |
| CG18039 | GluRIID | lethal | lethal |
| CG33976 | Octβ2R | sterile | fertile |
| Rescue Rate | | 6/9 | |
