# Supplementary material for: Conditional chemoconnectomics (cCCTomics) as a strategy for efficient and conditional targeting of chemical transmission
Source: eLife. 2024 Apr 30;12:RP91927. doi: 10.7554/eLife.91927 (PMC11060718; doi:10.7554/eLife.91927)
Supplement: Supplementary file 3. [file elife-91927-supp3.docx]

**List of sgRNAs targeting CCT genes**

| **CCTsgEF No.** | **CG No.** | **Spacer + PAM** | **CCTsgEF No.** | **CG No.** | **Spacer + PAM** |
| --- | --- | --- | --- | --- | --- |
| CCT1001sgEF | CG1171 | GGCCAGCAGCATGAAGAGCACGG | CCT1108sgEF | CG32447 | GATGGCCCCCAAACTAGCGCAGG |
| CCT1001sgEF | CG1171 | TTGACCTTCTCGCCGGATTGGGG | CCT1108sgEF | CG32447 | TGGGTGGACACTATACTGGGCGG |
| CCT1001sgEF | CG1171 | TGCAATGAGGACTTCGCTCTTGG | CCT1108sgEF | CG32447 | CACGCCCCGTCGGAGTTCAGGGG |
| CCT1002sgEF | CG11325 | GTATCTGTCCAGCTACGTGATGG | CCT1109sgEF | CG33639 | TCCCTCAGCGATTCCGTCCGCGG |
| CCT1002sgEF | CG11325 | ACACGGTGATGGACAATCGGTGG | CCT1109sgEF | CG33639 | GCCCAGATAAAGCTCCAGGTGGG |
| CCT1002sgEF | CG11325 | CTTGGTCAGCAGATACAGCACGG | CCT1109sgEF | CG33639 | CCGTCTTCAGCAGCGACCTGAGG |
| CCT1003sgEF | CG14375 | ACTGGTCGTTATCTGCACCGTGG | CCT1110sgEF | CG33696 | TGTATTGTATTCCGTGACATTGG |
| CCT1003sgEF | CG14375 | TTTCGCTTGGCTCTGTTGCGCGG | CCT1110sgEF | CG33696 | CTGCTCCGTCTGGTTTGTGGTGG |
| CCT1003sgEF | CG14375 | TCAGAGGGATGCCAGGCCTACGG | CCT1110sgEF | CG33696 | AGTGAGCGACACCTGCTTCCTGG |
| CCT1004sgEF | CG14593 | ACGGTGACAATGTACGTCTCCGG | CCT1111sgEF | CG34411 | GCCGGAACACTTCTTCCCCGAGG |
| CCT1004sgEF | CG14593 | GCCGTCGCTATCCGCCAGCCTGG | CCT1111sgEF | CG34411 | ACTGTGCCGTTGTATTGCAGTGG |
| CCT1004sgEF | CG14593 | CTCCTGCTCCAGACCTTGTCCGG | CCT1111sgEF | CG34411 | CTAAGCTCTCTGCTCCTAACGGG |
| CCT1005sgEF | CG4313 | GATGCACGGCACCATGAAGGCGG | CCT1113sgEF | CG43795 | GGAGAGCTCATTCGTGACGGCGG |
| CCT1005sgEF | CG4313 | GATCAGGAAAAGGTCAGCCTGGG | CCT1113sgEF | CG43795 | AATGCGACCGAGAGCGCCCGTGG |
| CCT1005sgEF | CG4313 | GATCGCAGGACGACCCTTTGTGG | CCT1113sgEF | CG43795 | GGAACCTGTCCTGAGCATACCGG |
| CCT1006sgEF | CG6371 | AGCAGGTAATAGGTCAGTGGCGG | CCT1114sgEF | CG44153 | GGTATGCTAAAGGCACGCCATGG |
| CCT1006sgEF | CG6371 | GGCACGTCAAAAGCGCGCCATGG | CCT1114sgEF | CG44153 | CCTCGGTGAGCGTGAAGACGCGG |
| CCT1006sgEF | CG6371 | TTGCACACTGCTGCTAATCGCGG | CCT1114sgEF | CG44153 | CGAAATAAACGAGACTACGCAGG |
| CCT1007sgEF | CG6936 | GCCATCAATTACCGGTCAGACGG | CCT1115sgEF | CG7497 | TTTGCATAGAGCGCTTTCGGAGG |
| CCT1007sgEF | CG6936 | GACCTACCGATGCCGTGTGACGG | CCT1115sgEF | CG7497 | ATGCATTGCAGCAGTTATGGCGG |
| CCT1007sgEF | CG6936 | TATCCATGATATGGTCGTGAGGG | CCT1115sgEF | CG7497 | CCACTCGGATGAACGACTGCAGG |
| CCT1008sgEF | CG31147 | AATGAATAGTGTCTCTCGCAGGG | CCT1116sgEF | CG8216 | GTTTGCGTCAGCACATACGGCGG |
| CCT1008sgEF | CG31147 | GCAAACTGAAGACATGCATCCGG | CCT1116sgEF | CG8216 | GATGCGGACGCCGCGAGCGAGGG |
| CCT1008sgEF | CG31147 | TAGTTCTTACCGGTACTTGTCGG | CCT1116sgEF | CG8216 | CTGGGCCTTGACGAATACCTGGG |
| CCT1009sgEF | CG17084 | GCTCCACGTGAGTGGACTCGCGG | CCT1117sgEF | CG12345 | AGGCAGTTCTCGGCTCCCCAGGG |
| CCT1009sgEF | CG17084 | TGAGTATTGCTTTAGCCCGTTGG | CCT1117sgEF | CG12345 | GGCCGACTATATCCGCGCCCTGG |
| CCT1009sgEF | CG17084 | CACTACTCCAGCGTATGAGTAGG | CCT1117sgEF | CG12345 | TTGAACAGGCCTATTACTACTGG |
| CCT1010sgEF | CG6496 | GTTGCGCTTCCTGATGGGCACGG | CCT1118sgEF | CG13936 | GATTGGACCGGGAGACGCCATGG |
| CCT1010sgEF | CG6496 | CCTCGACTGGTTCAACAACGTGG | CCT1118sgEF | CG13936 | GAGCAACAGATCCCTCAGCGAGG |
| CCT1010sgEF | CG6496 | TGGCGCCGCAGTATCCCCACTGG | CCT1118sgEF | CG13936 | CACAGGCTCGGCGATAATGGAGG |
| CCT1011sgEF | CG8784 | GTATTAAGTCGGAAACCGCCAGG | CCT1119sgEF | CG8380 | GACGAGCGCGAAACATGGAGCGG |
| CCT1011sgEF | CG8784 | GATACATATCCGGATACCAAAGG | CCT1119sgEF | CG8380 | CTTACGATTGTGCTGACCCAGGG |
| CCT1011sgEF | CG8784 | GCTCGCATTTGTGCCGTTCGTGG | CCT1119sgEF | CG8380 | GGTGCTGATAGCCTTCTATGTGG |
| CCT1012sgEF | CG13687 | GAGTCTGGAACACCGCGCGGAGG | CCT1120sgEF | CG9887 | CACGAAGAGATGCAGCGTGGCGG |
| CCT1012sgEF | CG13687 | GCCATTGCCATAATGGAAATGGG | CCT1120sgEF | CG9887 | TCTGACGGCGTTTAAGGAGAAGG |
| CCT1012sgEF | CG13687 | GTACATGGGCGGCAGCTGATGGG | CCT1120sgEF | CG9887 | TCATGATCGCCTTCGGCATGAGG |
| CCT1013sgEF | CG14734 | GCTGACGGCACCATCGAGTGCGG | CCT1121sgEF | CG5400 | GTGCGTATAATGACTTATGGCGG |
| CCT1013sgEF | CG14734 | CATCCAATGCGCCCTCTGAGCGG | CCT1121sgEF | CG5400 | TTTCTTGCAGACTCCATGGGTGG |
| CCT1013sgEF | CG14734 | GCCCGCTTTACCACCCGACGCGG | CCT1121sgEF | CG5400 | CTTGATTTTGTGCACCTTTGTGG |
| CCT1014sgEF | CG6515 | ATTGTGAACACCACGCTCCTGGG | CCT1122sgEF | CG18105 | TGCTCACTGCGTCCTATGCGAGG |
| CCT1014sgEF | CG6515 | CAACGTCACGGTCTCTACGTCGG | CCT1122sgEF | CG18105 | ACCAAGAACGTACCGCGGCTGGG |
| CCT1014sgEF | CG6515 | CAAGACCGTACGAGTTGCCATGG | CCT1122sgEF | CG18105 | CTGTCTGTTTCGCTCTTGGTGGG |
| CCT1015sgEF | CG7887 | ATGGTCATTGTGGCGACGGGCGG | CCT1123sgEF | CG5911 | GACTATGCAGAAGAACATGGCGG |
| CCT1015sgEF | CG7887 | GTCTTCCACGTCCCCGACGGCGG | CCT1123sgEF | CG5911 | CACGCCCACCGTCCTCGTGGAGG |
| CCT1015sgEF | CG7887 | GTTGAAGGTGACGTTCAGGCTGG | CCT1123sgEF | CG5911 | CATCTCGTGGCCAAGTACCCAGG |
| CCT1016sgEF | CG13633 | GACGAGATGGCCGACAACGGTGG | CCT1124sgEF | CG2346 | ACTTCATGCACTTCGGCAAGAGG |
| CCT1016sgEF | CG13633 | GCAGTAGGAGGTGGGCGTGAAGG | CCT1124sgEF | CG2346 | CGATGCCAATCACAGAGATGGGG |
| CCT1016sgEF | CG13633 | CAGCTCCCCGGTAATTGGCCAGG | CCT1124sgEF | CG2346 | GGCCGACTGCATCTGGTACAGGG |
| CCT1017sgEF | CG2872 | CGAATGATCCGTGTGTTCCGAGG | CCT1125sgEF | CG2114 | AATCGTCCTTAACCACACGGAGG |
| CCT1017sgEF | CG2872 | GACGAACAGAATGTCCGAGACGG | CCT1125sgEF | CG2114 | AGAACAATCGCATTGAGTTCTGG |
| CCT1017sgEF | CG2872 | TTGTGGATACGATCCGTTCGAGG | CCT1125sgEF | CG2114 | TGGATTGGCCCGCTGTGATACGG |
| CCT1018sgEF | CG10001 | ACTGGCCATATGGAAGGTTCTGG | CCT1126sgEF | CG15274 | GGATCTCGAGAATCGATGCATGG |
| CCT1018sgEF | CG10001 | GATTACGAACATCAGATCAGCGG | CCT1126sgEF | CG15274 | GCCTGCCGCAAGACTGGCGTTGG |
| CCT1018sgEF | CG10001 | AAGAAGAATCCCACTATCCACGG | CCT1126sgEF | CG15274 | TGCAGGATGAGCTTGAAGCCCGG |
| CCT1019sgEF | CG3302 | TCTTGCCGTTGGTCCATCCGCGG | CCT1127sgEF | CG6706 | GGCGTGGAAAACTCCTACACCGG |
| CCT1019sgEF | CG3302 | GAAGGTCTGGCCCATGCACATGG | CCT1127sgEF | CG6706 | GAAGGACGTGAGGATCATTCTGG |
| CCT1019sgEF | CG3302 | CCATCCGCGGGAGTACTGGAAGG | CCT1127sgEF | CG6706 | TCTACGTAATACATCCTTCGAGG |
| CCT1020sgEF | CG10698 | CTCCCCGATAATGCAGAACCAGG | CCT1128sgEF | CG14994 | CGAGTAATGGCACTGATCCGAGG |
| CCT1020sgEF | CG10698 | TGGTGCTTCATGGCGACTATTGG | CCT1128sgEF | CG14994 | ACGGGTATAAACTGTCCGAGAGG |
| CCT1020sgEF | CG10698 | GGTCAGCAATTGGTCCACACTGG | CCT1128sgEF | CG14994 | GCTTCATGTCCTCGGGATGGTGG |
| CCT1021sgEF | CG2902 | AATGGTGACCGAATCTACGCCGG | CCT1129sgEF | CG8442 | GCCAAAGCCAGAACGCAGGGGGG |
| CCT1021sgEF | CG2902 | ACGAGAGTACTCGATCGTGACGG | CCT1129sgEF | CG8442 | GTACACCTACGGAGAATCATTGG |
| CCT1021sgEF | CG2902 | GAATCCGCTTGTATAACTCACGG | CCT1129sgEF | CG8442 | ATCGATACCATCCAGTACTACGG |
| CCT1022sgEF | CG33513 | GGACGGGGACCTGCGCTCGGCGG | CCT1130sgEF | CG43743 | ATTCGTCCGGATACAGACCTCGG |
| CCT1022sgEF | CG33513 | CACTGTTAACAAGCTCCATGAGG | CCT1130sgEF | CG43743 | GCCGTGCAGCTTCTCACCAAAGG |
| CCT1022sgEF | CG33513 | GGAGGCCTACCTAACGGATCCGG | CCT1130sgEF | CG43743 | GGGGAACCAGGGCGTCACGAAGG |
| CCT1023sgEF | CG42301 | AGCTGTAACCGTCAGGACACCGG | CCT1131sgEF | CG6992 | AAGCTTGCTAATGAGATCCACGG |
| CCT1023sgEF | CG42301 | GCTCGGCGTATTCTGCATGCCGG | CCT1131sgEF | CG6992 | TCTGCACTACAAGAGTCCTCCGG |
| CCT1023sgEF | CG42301 | CTTAGGTCGGGATTGAGTGTCGG | CCT1131sgEF | CG6992 | ACGCGAGAGTACTGACAAGGAGG |
| CCT1024sgEF | CG32540 | GTAGTCGAGCAGTGCCCTATAGG | CCT1132sgEF | CG7234 | CCTTAATCCGTATAATGCCGAGG |
| CCT1024sgEF | CG32540 | TGGACTTGAACTGGTGCCCAGGG | CCT1132sgEF | CG7234 | GCATCCCCAGCAGTCGAACAGGG |
| CCT1024sgEF | CG32540 | GAGCAGGAACACGTTGGTTATGG | CCT1132sgEF | CG7234 | AGTCATATTATATCCAATCGAGG |
| CCT1025sgEF | CG33517 | CTACAATGGGAGCACGGTTTCGG | CCT1133sgEF | CG4226 | GCTGCGTTATCTGACCCCGAGGG |
| CCT1025sgEF | CG33517 | ACGGTGGCGCGCGAGACGCTGGG | CCT1133sgEF | CG4226 | CCTCTATCAAGGATATGCCGTGG |
| CCT1025sgEF | CG33517 | AGCACAAACTTGGCGGTGACGGG | CCT1133sgEF | CG4226 | AATGACCCTTAACCTCTACCCGG |
| CCT1026sgEF | CG13094 | GGGATATTCGGGTACCCAGGAGG | CCT1134sgEF | CG18039 | GATCACCTAATGACCCGATACGG |
| CCT1026sgEF | CG13094 | CTTGGCCATCTCGAGCATCGAGG | CCT1134sgEF | CG18039 | GGATGCAGATTGAAACCATCCGG |
| CCT1026sgEF | CG13094 | GCTTTGGACGCACCATCATACGG | CCT1134sgEF | CG18039 | GTCATTGCCACCGTCGCGGAAGG |
| CCT1027sgEF | CG32843 | GAGGTCTTCGAGATTTACGCAGG | CCT1135sgEF | CG31201 | GAGTGTTTGGACCCTCGTCGCGG |
| CCT1027sgEF | CG32843 | GTCCGGGCAGAGTTCGTAGGCGG | CCT1135sgEF | CG31201 | CCGGCGGGTTCGAAAGTCCGTGG |
| CCT1027sgEF | CG32843 | TCGCTGCCAATAACTCGTTGTGG | CCT1135sgEF | CG31201 | TCGCATGTAATTCTTGAAGGAGG |
| CCT1028sgEF | CG18090 | GACTAGTCTACAGAACGCTAAGG | CCT1136sgEF | CG14723 | GATTTGCGTGTCATGCGGATAGG |
| CCT1028sgEF | CG18090 | CATTCTCTCTATTCGGGGACAGG | CCT1136sgEF | CG14723 | CCAGGGAGAGGACCGTGACATGG |
| CCT1028sgEF | CG18090 | CACGCTGTTTATGCCACTCTGGG | CCT1136sgEF | CG14723 | GGCAATATATTTGCACGCGCTGG |
| CCT1029sgEF | CG6440 | CAGTTGATCGGAGTTCTCCAGGG | CCT1137sgEF | CG13586 | CCGACTGGACCGCATCTGCGAGG |
| CCT1029sgEF | CG6440 | TCCTGGCCGTGTCCAACACACGG | CCT1137sgEF | CG13586 | TTCTTCGACCTGGAGTGCAAGGG |
| CCT1029sgEF | CG6440 | GCTTGTTGTAGTTCTTCAACAGG | CCT1137sgEF | CG13586 | CCACCGAGATCTTTATGTTGCGG |
| CCT1030sgEF | CG43745 | ATGCCGGTGCCGCAATAATGCGG | CCT1138sgEF | CG7665 | CGATGTCTACCCCAATCTCACGG |
| CCT1030sgEF | CG43745 | GGCAGTTTGGCGGTACATAGCGG | CCT1138sgEF | CG7665 | CTGCTGCTAACGTCGCTGAGTGG |
| CCT1030sgEF | CG43745 | CTCGCGGCAGCCTTACACTGAGG | CCT1138sgEF | CG7665 | AGCACCCGAGTCTGTCCCAGCGG |
| CCT1031sgEF | CG8348 | GTTCCATCCGGGTAGTCCAGAGG | CCT1139sgEF | CG31096 | GTGACCTCCGAACTGAGACGCGG |
| CCT1031sgEF | CG8348 | GCTGTCCATTGTCAATCCGCTGG | CCT1139sgEF | CG31096 | GGTCATCAGACAGAAGCCTACGG |
| CCT1031sgEF | CG8348 | GGCCGTGCAGACGAGCCTTGTGG | CCT1139sgEF | CG31096 | TAGAGGAGACCGCACTCCACCGG |
| CCT1032sgEF | CG8422 | CTGCACAACCTAGATGGCATCGG | CCT1140sgEF | CG7918 | TGTATCATTCTTACGGTGGGCGG |
| CCT1032sgEF | CG8422 | GCCACTATCGTTGCCATAGGTGG | CCT1140sgEF | CG7918 | CAAGCGAGTCCTGACGCACACGG |
| CCT1032sgEF | CG8422 | AGGGTACCGCGAGCTGTCCTCGG | CCT1140sgEF | CG7918 | GCTCAAGGGTTATTGGGACCTGG |
| CCT1033sgEF | CG12370 | GCTGTTGACACTGTAGCGTGCGG | CCT1141sgEF | CG11144 | GTTGAGGCCGGTGATACTTGAGG |
| CCT1033sgEF | CG12370 | AGACGACGATTTGAGGGCACTGG | CCT1141sgEF | CG11144 | GCGACCAAACGACTGAGACAAGG |
| CCT1033sgEF | CG12370 | TAGTGATCCCACGTTCCGTTTGG | CCT1141sgEF | CG11144 | CAAAAGAAGCCTAACGCTCGCGG |
| CCT1034sgEF | CG13480 | CAGCGATTCCACTCGTGGGGCGG | CCT1142sgEF | CG4322 | TATCCAATACGGAAACATAGGGG |
| CCT1034sgEF | CG13480 | GGCTGGCTCCATAGACTTGACGG | CCT1142sgEF | CG4322 | GGCCCTGCTGAAATGTCCCAAGG |
| CCT1034sgEF | CG13480 | GGTGGGGCGTAGTGCCGGAAGGG | CCT1142sgEF | CG4322 | GGAGGACGGCTATCCCCCCCTGG |
| CCT1035sgEF | CG10626 | GGAATTCCTGCCCGGAGCCGAGG | CCT1143sgEF | CG8985 | GATATTCAAGGTATTCGCGATGG |
| CCT1035sgEF | CG10626 | TACACTCAGGGCCTGGACGAAGG | CCT1143sgEF | CG8985 | GAAACTGAGCCGCTCTACTGCGG |
| CCT1035sgEF | CG10626 | CTATGGGGGAATCAGTATCGTGG | CCT1143sgEF | CG8985 | GATGTAGTCGTGTATGGTGTAGG |
| CCT1036sgEF | CG4128 | GTGCTCTTGAAGATACCAGGGGG | CCT1144sgEF | CG17061 | GCACAGCGAGGAGTAACCCACGG |
| CCT1036sgEF | CG4128 | CAATACGCTGGAGCGACCCGTGG | CCT1144sgEF | CG17061 | CCTGGAGGATCCCTATACTGCGG |
| CCT1036sgEF | CG4128 | AACGGAATACGGCGGGGTCAAGG | CCT1144sgEF | CG17061 | GAACGGATCCATGCTCCGTGTGG |
| CCT1037sgEF | CG33976 | GCCCGGAGCCACCGCGGCAAAGG | CCT1145sgEF | CG32853 | GCTGACGGATCCAACTTTAGCGG |
| CCT1037sgEF | CG33976 | AACATTGGCGCGGGTCACGGCGG | CCT1145sgEF | CG32853 | TCTTTATCCCGAAATCTACTCGG |
| CCT1037sgEF | CG33976 | CAACATCGTTTGGGTGTTCAAGG | CCT1145sgEF | CG32853 | GAAATTCCTGCCAACCTGACCGG |
| CCT1038sgEF | CG13758 | TCTACGCCATGAAAGCCGCCAGG | CCT1147sgEF | CG30018 | ACTCGATGATAATTCGACAGAGG |
| CCT1038sgEF | CG13758 | GCATCGAAATCGTGCAGTAGTGG | CCT1147sgEF | CG30018 | CTTGTGTGATATAATCCATATGG |
| CCT1038sgEF | CG13758 | GACGCAAGCGTTAGATTCCAGGG | CCT1147sgEF | CG30018 | TATGGATGAACTACGATTGTTGG |
| CCT1039sgEF | CG14358 | GCTCATGGCAAGCGGTCCGGCGG | CCT1148sgEF | CG17795 | GATACCGTTGATATCTCGGAAGG |
| CCT1039sgEF | CG14358 | GAATACGGACATTCGTGTTGGGG | CCT1148sgEF | CG17795 | GGTGCAAAGGCACTATAAGAAGG |
| CCT1039sgEF | CG14358 | TCAGGTTCCTGCCTGGAATACGG | CCT1148sgEF | CG17795 | TCATTAGCTTCTTAACGCACAGG |
| CCT1040sgEF | CG30106 | GAGACACCCTACGTGCCCTACGG | CCT1149sgEF | CG6536 | GTGATTGCAGTCATGATTAGCGG |
| CCT1040sgEF | CG30106 | CATCCTTCATGAACTCCGACAGG | CCT1149sgEF | CG6536 | GCATACAACTTATACGCCTGGGG |
| CCT1040sgEF | CG30106 | TTCGCATCTGTCGCACACTGAGG | CCT1149sgEF | CG6536 | GACTAAACTTAATCCGCAAGTGG |
| CCT1041sgEF | CG13575 | GCTCGAGTTGTACCTAGAATGGG | CCT1150sgEF | CG6965 | CCGATGTTCACCAGCTATGGAGG |
| CCT1041sgEF | CG13575 | CCAGTGAGATGAGGCAGGCCCGG | CCT1150sgEF | CG6965 | ACCAGTCATGTCACTAGCGCCGG |
| CCT1041sgEF | CG13575 | CCCTCGATCCAGCCGTATCCGGG | CCT1150sgEF | CG6965 | TGGCCAATATACCACTATGCTGG |
| CCT1042sgEF | CG13995 | TCTAGGCAATCCGTATGACGTGG | CCT1151sgEF | CG16992 | GAATTCGGATCTTCCAAACGGGG |
| CCT1042sgEF | CG13995 | TTGCCATAGTCGTAGTCCGCGGG | CCT1151sgEF | CG16992 | GATTAGCTCCTCGTACGCATAGG |
| CCT1042sgEF | CG13995 | GAGGGCGAGATGCAGCTCCGCGG | CCT1151sgEF | CG16992 | GAAGGTTGTTTCGGTACAGGAGG |
| CCT1043sgEF | CG33495 | CTAAACTTAGGACCCTACCTCGG | CCT1152sgEF | CG7476 | TGAGTGGTACTCGTGCCTGTAGG |
| CCT1043sgEF | CG33495 | AGGATCGAAATGATACGGAGTGG | CCT1152sgEF | CG7476 | CTAAGGATCCAGGAGATATGTGG |
| CCT1043sgEF | CG33495 | ACGACCAAGAGGAGAAATAGCGG | CCT1152sgEF | CG7476 | GTGTCGTAGTAGTTGCACCCGGG |
| CCT1044sgEF | CG31720 | GGTTCCATCGATCTTGCAATAGG | CCT1153sgEF | CG32475 | GCAAACATAACGGGGAGCTACGG |
| CCT1044sgEF | CG31720 | TACGAATACTCAGTACTGCGTGG | CCT1153sgEF | CG32475 | GAGGCAGTGCTTAGTCCCTGTGG |
| CCT1044sgEF | CG31720 | AAACGTAAATAAATCCCTGTGGG | CCT1153sgEF | CG32475 | GATGGTCTACCTTATGTGTTCGG |
| CCT1045sgEF | CG30361 | ATGCACCGCGACCTGTGCGGCGG | CCT1154sgEF | CG12414 | ACATCTGTCGTATTAACAACAGG |
| CCT1045sgEF | CG30361 | AGTACACCGAGGGCGAGCTGCGG | CCT1154sgEF | CG12414 | AATATAATAATACCGTGCATGGG |
| CCT1045sgEF | CG30361 | CATCCACTTCAAGCAGTCCCAGG | CCT1154sgEF | CG12414 | TTGGCAGTTGGACTTATGATGGG |
| CCT1046sgEF | CG10342 | GCCGGCGGCTAGGAGGGCAAGGG | CCT1155sgEF | CG32975 | CGTGCTGATGTACAACAGTGCGG |
| CCT1046sgEF | CG10342 | TTGACATCGTTCTTTCGCGGAGG | CCT1155sgEF | CG32975 | AATACTGAACTCATTGTCGCTGG |
| CCT1046sgEF | CG10342 | GACAGAGCCCGCGTTCGGTTCGG | CCT1155sgEF | CG32975 | GTCATTGCTTCTACCGTGGTTGG |
| CCT1047sgEF | CG1147 | TGGACCCGGTGCTTATCGATAGG | CCT1156sgEF | CG32538 | ATCTCGTTCTATCCTGACCAGGG |
| CCT1047sgEF | CG1147 | GCTGATGAGCATGTGGTACCAGG | CCT1156sgEF | CG32538 | TTGGTTCGTGGACCTACGATGGG |
| CCT1047sgEF | CG1147 | GCGTGCGCTATCTGGACGACCGG | CCT1156sgEF | CG32538 | TGGAGTAGCCGCTTCTCATGGGG |
| CCT1048sgEF | CG13061 | AGAGGAGATCCGACAACGGTGGG | CCT1157sgEF | CG11822 | GGACGCGGGTGTGTAGATGACGG |
| CCT1048sgEF | CG13061 | CAGAGGAGAAGGATGCACCTGGG | CCT1157sgEF | CG11822 | GATCGGCTCCTGGGGCCTGAAGG |
| CCT1048sgEF | CG13061 | GATCAAGAGCGTCCATGGCCTGG | CCT1157sgEF | CG11822 | AGTTCGTGAACAGGCCGGCGTGG |
| CCT1049sgEF | CG8795 | GGTGTCATCGGGGTCACCGTGGG | CCT1158sgEF | CG34388 | AGGCGGCCTTGAGCCACCAACGG |
| CCT1049sgEF | CG8795 | AAATCATGTCGGATATAGCGAGG | CCT1158sgEF | CG34388 | GGAGGAGCTGGCCCCAGAATCGG |
| CCT1049sgEF | CG8795 | GGAGAGCGTTCTCTCGGAAACGG | CCT1158sgEF | CG34388 | GCTGGCCCCAATACCGATGAGGG |
| CCT1050sgEF | CG16752 | GATCCGCGTCAGATGGTCCGCGG | CCT1159sgEF | CG3441 | GCACTCACAATAATGGACCGTGG |
| CCT1050sgEF | CG16752 | GGCATACAGTAGCCATAGAGCGG | CCT1159sgEF | CG3441 | GGAGGAGGACAAACGTTCGGTGG |
| CCT1050sgEF | CG16752 | GCTCAAAACGAGCACAATCAGGG | CCT1159sgEF | CG3441 | GAAGCTGGTACTCGGGACTGGGG |
| CCT1051sgEF | CG14871 | TTATGGCCTGCGAACTGGGGGGG | CCT1160sgEF | CG11051 | GAATTCGTTGAAATCACCCTGGG |
| CCT1051sgEF | CG14871 | GTAGTTAAAGCAGCATGTGCTGG | CCT1160sgEF | CG11051 | CAGGATTGGATTCCTCACGGGGG |
| CCT1051sgEF | CG14871 | TGACTAAGACAACGATGCATTGG | CCT1160sgEF | CG11051 | GTCCGCCCGCGTCCCCCGTGAGG |
| CCT1052sgEF | CG34381 | TCCGTCCCCCGAGAGTCCGGTGG | CCT1161sgEF | CG15361 | GATTGGGGCGGGATTGGCACGGG |
| CCT1052sgEF | CG34381 | CCACCGGACAACAACCGCCCCGG | CCT1161sgEF | CG15361 | CTAGCTCCGTAGTAGTACTGGGG |
| CCT1052sgEF | CG34381 | AGTGGACAAACTGGTACATGCGG | CCT1161sgEF | CG15361 | AGCAAACAACATGTTCAAGCTGG |
| CCT1053sgEF | CG16720 | GGAGCGGAATCTACAAAACGTGG | CCT1162sgEF | CG3856 | GATGAACTCGAGTACGGCCAGGG |
| CCT1053sgEF | CG16720 | GGCCACCAGATGCAAAATCGAGG | CCT1162sgEF | CG3856 | GGCCAAACCCACCAGAAGATCGG |
| CCT1053sgEF | CG16720 | TGGTCCTGACCATCTACATTCGG | CCT1162sgEF | CG3856 | TCTCTGGTGCCGCATTTGGCTGG |
| CCT1054sgEF | CG15113 | TGGTCGATGACGAACAAGAGCGG | CCT1163sgEF | CG7485 | GGCGGCGGCTCTAACCGCTGCGG |
| CCT1054sgEF | CG15113 | TCTATTTCCAAAATGCTCGCTGG | CCT1163sgEF | CG7485 | ACTCAATGCCGCGGTAACGGTGG |
| CCT1054sgEF | CG15113 | AATGGCCACCAGGTGCAGTATGG | CCT1163sgEF | CG7485 | CAACGTGGCTTACTCGATCCTGG |
| CCT1055sgEF | CG12073 | CAGCTGGATAGCCTATGCGGCGG | CCT1164sgEF | CG13565 | GCGTTAAGAGAATTGTGTAACGG |
| CCT1055sgEF | CG12073 | GCAAATTCCTCCAGGAGCAAAGG | CCT1164sgEF | CG13565 | GGAACACCGATACCACCGCCAGG |
| CCT1055sgEF | CG12073 | TCCGTCTCGCCTTCCACGAGCGG | CCT1164sgEF | CG13565 | CATTCACGCAGCGCCCGGTGTGG |
| CCT1056sgEF | CG14919 | GGTCCTGTTTCGGCACCCGAAGG | CCT1165sgEF | CG15284 | GAACTGCGAGACTCTCAAGTCGG |
| CCT1056sgEF | CG14919 | CCTCGCTGAGGGCAAAGAACAGG | CCT1165sgEF | CG15284 | AGGGCATTGCGATATAGCCAAGG |
| CCT1056sgEF | CG14919 | GACGTGCGAGGCGCCTATGGAGG | CCT1165sgEF | CG15284 | GCATTGGGGCACTAGGATCGCGG |
| CCT1057sgEF | CG14575 | TCCTCTCCAAATCCATGGGATGG | CCT1166sgEF | CG31660 | CGATGAGCCGACACGTGCGGAGG |
| CCT1057sgEF | CG14575 | GGTCTGTGGTCCAAGCACAAAGG | CCT1166sgEF | CG31660 | TGTGTCCGCTCTCAGTCGGGCGG |
| CCT1057sgEF | CG14575 | TAAGAGATTACCAACGACCCCGG | CCT1166sgEF | CG31660 | CAACATTATCCGAATCCACTAGG |
| CCT1058sgEF | CG33344 | TGCGATATCACACGTGATGCTGG | CCT1167sgEF | CG7105 | GCTGTGGAAGTGGACACAGGTGG |
| CCT1058sgEF | CG33344 | GGACGCCAGCCCCGAGGATTCGG | CCT1167sgEF | CG7105 | GCGACCTTGTCGGCAGGTAACGG |
| CCT1058sgEF | CG33344 | GTCAAATTCGCCGGGGTCGCTGG | CCT1167sgEF | CG7105 | CTCCACAATGAGGCCACCGGTGG |
| CCT1059sgEF | CG13579 | GTATGTAGGCCGCATAGCGGCGG | CCT1168sgEF | CG6986 | AGTCCAATAACAAAAACGCACGG |
| CCT1059sgEF | CG13579 | GGAAAGCGCCCCTCGGAGCAGGG | CCT1168sgEF | CG6986 | GTAGAGCTTGAAATGATACTTGG |
| CCT1059sgEF | CG13579 | TGACAGAAGATCTCTCCGTATGG | CCT1168sgEF | CG6986 | GATTAACTGGCACGTCAGGTAGG |
| CCT1060sgEF | CG31760 | GATAGGGCTCCGCCTCGCATTGG | CCT1169sgEF | CG10537 | GGTGTAGAAACACTATCGGTTGG |
| CCT1060sgEF | CG31760 | GCCCACTTCCTGGAGTATCGAGG | CCT1169sgEF | CG10537 | ACGGATCCATACAGTGCAAGCGG |
| CCT1060sgEF | CG31760 | GTCCTACGTCTGGTGGGTCAAGG | CCT1169sgEF | CG10537 | TTTGTCGTAACTAACACTAAAGG |
| CCT1061sgEF | CG32547 | GTAGGGCGAGAGCTGCGTACTGG | CCT1170sgEF | CG8930 | GAAAACCTCCCAGGTCCAGGAGG |
| CCT1061sgEF | CG32547 | GTTGACATGGAGCGACTTAGTGG | CCT1170sgEF | CG8930 | AAGTCCTTGCAGGAATACCATGG |
| CCT1061sgEF | CG32547 | CAGATTGATGATGAACGCATGGG | CCT1170sgEF | CG8930 | GCGCAGGGTAGAAAAGGTCCTGG |
| CCT1062sgEF | CG18314 | GCAGGAAATGAGCTACCTACAGG | CCT1171sgEF | CG40733 | AGAAAGAAATGCTCGTTTCGAGG |
| CCT1062sgEF | CG18314 | GTAGTAGTTGATCACCTCTGTGG | CCT1171sgEF | CG40733 | TTCTTTCTTGGCTCTCGGTACGG |
| CCT1062sgEF | CG18314 | TATCGGTATACACCTTCATGTGG | CCT1171sgEF | CG40733 | AGGTACGATGAAAATGCGTCTGG |
| CCT1063sgEF | CG9652 | GGATGTTTCCGGCCACCGAAAGG | CCT1172sgEF | CG5811 | CGACTATGACCTTCTATCGGAGG |
| CCT1063sgEF | CG9652 | AATCACACCAGTGGGAGCACCGG | CCT1172sgEF | CG5811 | GACCGTGCGCATGCGAGGTGTGG |
| CCT1063sgEF | CG9652 | CGTCGAGCACATGACATCAAAGG | CCT1172sgEF | CG5811 | GACGATGAAGGGTCCAACTATGG |
| CCT1064sgEF | CG3022 | ATACGGTCCATGGCCGGCCGTGG | CCT1173sgEF | CG4545 | GAGAGGACTCGCGAGACCTGGGG |
| CCT1064sgEF | CG3022 | CGTCCGTTTGTACTTCCAATCGG | CCT1173sgEF | CG4545 | GGGATTCGAGCGGCCCGTCGTGG |
| CCT1064sgEF | CG3022 | GGCCCTTGCCTGGGCAACGTCGG | CCT1173sgEF | CG4545 | TGGACTGCCCCTGTTCTACATGG |
| CCT1065sgEF | CG3454 | CTATATGCGCCAGTTACTGCCGG | CCT1174sgEF | CG33527 | AAGATGCTGCCGTTGAACGGAGG |
| CCT1065sgEF | CG3454 | GACAGTACTCACCCCTTTGGCGG | CCT1174sgEF | CG33527 | GCTCCAGTGAGGCAGCCTACAGG |
| CCT1065sgEF | CG3454 | GCAGGCCGGCGAGCTCGCCCAGG | CCT1174sgEF | CG33527 | CTGCGACCAGGATCGTGACCAGG |
| CCT1066sgEF | CG4395 | TGACTCAGCACAGTGCCGGCCGG | CCT1175sgEF | CG10823 | GCTGGGCACGGTTCTCACTACGG |
| CCT1066sgEF | CG4395 | GCGGCATTCTCCAGATATCGCGG | CCT1175sgEF | CG10823 | CCATTCCGACCATGGCGCCGGGG |
| CCT1066sgEF | CG4395 | CCAGAATGTCACATACGATACGG | CCT1175sgEF | CG10823 | GTAGGCCACGCAGTAGACCATGG |
| CCT1067sgEF | CG4356 | CGGGCTATACGATCCCTATTCGG | CCT1176sgEF | CG3171 | ATGCGGGTAGATCGATTGCGTGG |
| CCT1067sgEF | CG4356 | GCCAATGCCAGACTCATGACCGG | CCT1176sgEF | CG3171 | ACATTGCCATAGAAGATCACCGG |
| CCT1067sgEF | CG4356 | AGCCAGGTGTCGCAGACGATGGG | CCT1176sgEF | CG3171 | GCTCAAGAGCCCCACGATACGGG |
| CCT1068sgEF | CG6456 | CTATGGCTCACACTAAGACGCGG | CCT1177sgEF | CG7431 | GTAGGAGGTGGATATCCCAGCGG |
| CCT1068sgEF | CG6456 | CCACCCTCTAATGAACCAGGAGG | CCT1177sgEF | CG7431 | GTAGAAGTGCGCCCATTCGGCGG |
| CCT1068sgEF | CG6456 | ACGGCAACAATAAGCGCGCCTGG | CCT1177sgEF | CG7431 | GAAGATGCCCACGAGAAGGTCGG |
| CCT1069sgEF | CG4521 | GGATCCTCCAGAGCATTGATCGG | CCT1178sgEF | CG16766 | GGCGTGTGGTCAGAACGGCGAGG |
| CCT1069sgEF | CG4521 | GCATGGTGATAGCAAGCGTATGG | CCT1178sgEF | CG16766 | CAGACCAGTGCGGCACCAGGTGG |
| CCT1069sgEF | CG4521 | GGCAGCTATCAGACTAGCTACGG | CCT1178sgEF | CG16766 | GGATGGAGCCGCTGCAGAGCAGG |
| CCT1070sgEF | CG6530 | GCCGCTAAATGAATTCCAGAGGG | CCT1179sgEF | CG8394 | CATTCCGGAACGCCGGTAGCTGG |
| CCT1070sgEF | CG6530 | GAAATATCCCAGGAACAAGGAGG | CCT1179sgEF | CG8394 | AAAGACTACGAGCAACCGCCGGG |
| CCT1070sgEF | CG6530 | CATTTAGCGGCAACTCCAGCTGG | CCT1179sgEF | CG8394 | CCTTCAATGAGTACGACGGCAGG |
| CCT1071sgEF | CG5610 | GCAGAAGGATTTGTAAATGGCGG | CCT1180sgEF | CG3822 | GCGAGGTCACCAGGTAGCTATGG |
| CCT1071sgEF | CG5610 | GACCGTCTCACCGTCAAGATGGG | CCT1180sgEF | CG3822 | GGACATTGTCGAGCTCACTCCGG |
| CCT1071sgEF | CG5610 | ACTACTACATCTCCGTGGAGTGG | CCT1180sgEF | CG3822 | TCCCCACTTGGAGAATCGCTGGG |
| CCT1072sgEF | CG6844 | GTGACGCCGCCATACTCCGAGGG | CCT1182sgEF | CG5549 | ATAGACGGCCACGGGACCCAGGG |
| CCT1072sgEF | CG6844 | GGATACGGTTCGGCACAGCAGGG | CCT1182sgEF | CG5549 | TCGCTGCTCGGATACTCAGTGGG |
| CCT1072sgEF | CG6844 | TCGCCTGAACGGTTTCGCAGAGG | CCT1182sgEF | CG5549 | AGGCGTGGTCTGCCCCTCTACGG |
| CCT1073sgEF | CG2302 | GAGCTCCTGCGAAATCGACGTGG | CCT1183sgEF | CG5621 | GATGCTCATCGCGTGACGGGCGG |
| CCT1073sgEF | CG2302 | GTCCGGACGCCAGATGTGATCGG | CCT1183sgEF | CG5621 | GGTCTCCTTTAGCATGCCATAGG |
| CCT1073sgEF | CG2302 | TTCGACGGACGTATAGAACTCGG | CCT1183sgEF | CG5621 | CAAACCGAATGGGTCCCGTGAGG |
| CCT1074sgEF | CG11348 | GGCTTCCAAACCTTGTCGGGGGG | CCT1184sgEF | CG7446 | GATCCGGGGCCGATGGACCAGGG |
| CCT1074sgEF | CG11348 | ATGTGATGTCAGTCTCCGTGGGG | CCT1184sgEF | CG7446 | GGTGGCTCGGGGTCGACCACCGG |
| CCT1074sgEF | CG11348 | ATAACGGCCCTTGCCCGAAAGGG | CCT1184sgEF | CG7446 | CGTCGGATGTCATCTACCGTTGG |
| CCT1075sgEF | CG6798 | GGAGTATTACCCCGATACACTGG | CCT1185sgEF | CG7589 | GTCTTTCCAGTCGATCATAGCGG |
| CCT1075sgEF | CG6798 | TTCTCCCGTATACTTCAGAGTGG | CCT1185sgEF | CG7589 | GATCATCTCCACGCGCATGCAGG |
| CCT1075sgEF | CG6798 | ACTTGCACTAGGTTACTGCCAGG | CCT1185sgEF | CG7589 | TCGTAGTGTTGTGCCAGAACTGG |
| CCT1076sgEF | CG6919 | GACCACGTCTTCAACGGATACGG | CCT1186sgEF | CG8681 | GGTCGAGGCCAACATAACTACGG |
| CCT1076sgEF | CG6919 | TTCGTCAAGTGTTTCATTATTGG | CCT1186sgEF | CG8681 | TTTATTGTATCCTCACGCCTGGG |
| CCT1076sgEF | CG6919 | TAATGCTTCCGTCATGATCTCGG | CCT1186sgEF | CG8681 | ACAGTAAAACAACTGGGGCCGGG |
| CCT1077sgEF | CG9918 | GGCCGTAATCGTTAGCACCGTGG | CCT1187sgEF | CG9935 | GAACTTCTCCGTGAAATGACCGG |
| CCT1077sgEF | CG9918 | GGCCATCGTGATACCCGTAACGG | CCT1187sgEF | CG9935 | GGGCAGACCGGCCTATGTTTGGG |
| CCT1077sgEF | CG9918 | CCTGCGGAACGCCCGACAACAGG | CCT1187sgEF | CG9935 | CCAGCCTGGAATGTATAACTCGG |
| CCT1078sgEF | CG7395 | GGACATTTCCGAAGACACCCAGG | CCT1188sgEF | CG11155 | TCTTCTTTAACCATAACATACGG |
| CCT1078sgEF | CG7395 | ATGGCACAGACTCCTGCCGAAGG | CCT1188sgEF | CG11155 | ATCATTCCAGGTTTGGTCGGAGG |
| CCT1078sgEF | CG7395 | CAACTGGAGCCTAACGTCGCCGG | CCT1188sgEF | CG11155 | CTAGCTAAAAGAGCATCTGTTGG |
| CCT1079sgEF | CG11895 | GTCTCCGTGAATCACAAGGATGG | CCT1189sgEF | CG12344 | TTAGGCGGAAGAACTACTCGCGG |
| CCT1079sgEF | CG11895 | TTTAAGGTAGATTCCAGGACAGG | CCT1189sgEF | CG12344 | CTACGTGACGCTGCTTCCAGAGG |
| CCT1079sgEF | CG11895 | GAGATCCGTCGATGCAGGGCGGG | CCT1189sgEF | CG12344 | CGAAGCGGAAGTATACGGTGAGG |
| CCT1080sgEF | CG9122 | GTTTCCCACCTGGTTACGCAGGG | CCT1190sgEF | CG32848 | GGGCTATCGATACAATCACGAGG |
| CCT1080sgEF | CG9122 | CTACACTTCCGTGAACACGCAGG | CCT1190sgEF | CG32848 | CCCGTGATATTATCCCTCAACGG |
| CCT1080sgEF | CG9122 | CAGGCCCAGAAGACTCTTCCCGG | CCT1190sgEF | CG32848 | TCGACGGCAGTCTTTGCTTGTGG |
| CCT1081sgEF | CG1056 | TTTCTGGACTACAGTCCCCACGG | CCT1191sgEF | CG7535 | GCCTTCGTATAGGAAACGGGCGG |
| CCT1081sgEF | CG1056 | ACTGTTGCGATGGCCGTACGAGG | CCT1191sgEF | CG7535 | GAGCAGATCTGTCTATCCAGCGG |
| CCT1081sgEF | CG1056 | ATGCAAAGCTACTCTGGTCGTGG | CCT1191sgEF | CG7535 | AAAGTATCTGACCCTGACGGAGG |
| CCT1082sgEF | CG42796 | GCACCTGGTCCTGTGGGAGGAGG | CCT1192sgEF | CG17336 | GAGGCGCTGATAGACTCCAGTGG |
| CCT1082sgEF | CG42796 | CATCCAGGCAGATCCAGGTGAGG | CCT1192sgEF | CG17336 | GTAGTGCAGATCCATCATGCAGG |
| CCT1082sgEF | CG42796 | TCGTCCTGGGAACAGCGGCCGGG | CCT1192sgEF | CG17336 | GTATTGGCGCGACGAGCGTTTGG |
| CCT1083sgEF | CG9753 | GTTTCTGGGTAGTCCCATGGAGG | CCT1194sgEF | CG11236 | ACTGGCAACACCATTAACCCCGG |
| CCT1083sgEF | CG9753 | GGTGTAGGGTATGTTCAGCTCGG | CCT1194sgEF | CG11236 | TGGGGTCCGTACCTTCTGGGCGG |
| CCT1083sgEF | CG9753 | GGCGTCGCAACTTTCTTTCCCGG | CCT1194sgEF | CG11236 | CATAAAGGTGACGATCATATCGG |
| CCT1084sgEF | CG18208 | CGTCTTCTCGACCGGAAAGCCGG | CCT1195sgEF | CG12338 | CTCCTTCTGGAGTTCTAAGGCGG |
| CCT1084sgEF | CG18208 | TCGCTGGCCAATGAGCTAATGGG | CCT1195sgEF | CG12338 | TAGTGGCATAGGGCAAGAAGAGG |
| CCT1084sgEF | CG18208 | CGAATCACACAGAAGTCCACTGG | CCT1195sgEF | CG12338 | ATCCCAGTAGTTGAAGGCATCGG |
| CCT1085sgEF | CG18741 | AGGAGCCGGCGCGCCCCTCCTGG | CCT1196sgEF | CG4827 | GGTGCCCTTCCTAAATGCCGTGG |
| CCT1085sgEF | CG18741 | CCGTGTGCAAGTACCGCTCCCGG | CCT1196sgEF | CG4827 | GAATCCCATTGCACCCCGGGCGG |
| CCT1085sgEF | CG18741 | CCTCCTGTTGCCACCATCGCAGG | CCT1196sgEF | CG4827 | GTTCCGAAAGGCAAGCACCTTGG |
| CCT1086sgEF | CG32476 | AGACAACCCGTTAGATCCGGCGG | CCT1197sgEF | CG30104 | TATCGTAAGGAGGCTCAGGAGGG |
| CCT1086sgEF | CG32476 | GCAAATAACTAACTAATAGCAGG | CCT1197sgEF | CG30104 | TCAGTTGGGGAACCTTGCTTAGG |
| CCT1086sgEF | CG32476 | CAACGAATCATACACTGTGAAGG | CCT1197sgEF | CG30104 | TGGCCACTCGGGTTATTTGAAGG |
| CCT1087sgEF | CG42244 | CATTGCGGTGGATAACCAGGCGG | CCT1198sgEF | CG8129 | GGCCATGCGAATGTCCCGCGAGG |
| CCT1087sgEF | CG42244 | GCCTATAACATCGCAATTTGTGG | CCT1198sgEF | CG8129 | ATCTATTAGCGGCATTGCGGTGG |
| CCT1087sgEF | CG42244 | AACGCCTCCGTGGAACTGTCCGG | CCT1198sgEF | CG8129 | TTTAACGGCTGTGGCAATACCGG |
| CCT1088sgEF | CG7411 | GGAATACCGCCTGCTCGAGGTGG | CCT1199sgEF | CG3011 | GGCCTGGATCTGCCCGATGGCGG |
| CCT1088sgEF | CG7411 | GGGGCGTTGTTGGATCCCACTGG | CCT1199sgEF | CG3011 | AGGGATATCCCGGCAAGAGGTGG |
| CCT1088sgEF | CG7411 | GTTCACCCTTAAGCGTCGTTTGG | CCT1199sgEF | CG3011 | GTCTCCGGGTTCACTTTGTACGG |
| CCT1089sgEF | CG1543 | GGTGAGACGGGACTACCAGCAGG | CCT1200sgEF | CG13317 | GCCGCTGATACCCACGCCGGAGG |
| CCT1089sgEF | CG1543 | CAATGTGACGGCACCGCACGAGG | CCT1200sgEF | CG13317 | TTTTGTTGCGCCGTGTCAGTCGG |
| CCT1089sgEF | CG1543 | TGTGATGATGATAAGCCAGTTGG | CCT1200sgEF | CG13317 | AGTTGACAACTCACCGCTCGCGG |
| CCT1090sgEF | CG10118 | GGAGTCCCGTCAGTCGCGCGTGG | CCT1201sgEF | CG14049 | GATATGCGTAAGCGGAACGGTGG |
| CCT1090sgEF | CG10118 | GAGGCGTGCTTGGGGAACCACGG | CCT1201sgEF | CG14049 | TATCACATCGCTGAGGCCGGTGG |
| CCT1090sgEF | CG10118 | ATCCCGCCGTCGCAGCCTGGTGG | CCT1201sgEF | CG14049 | GGCCACCTTGTTCGCCGTGGCGG |
| CCT1091sgEF | CG7285 | TGTCCTCCAGTTTGTGAAAGGGG | CCT1202sgEF | CG14059 | GTGCGAGCACCTCTTTCAGGCGG |
| CCT1091sgEF | CG7285 | GCTATGCAATGTTGATAGGTGGG | CCT1202sgEF | CG14059 | ACTCGATGGACCTTCTGTCGCGG |
| CCT1091sgEF | CG7285 | ATATATCCTGAATCTGGCGGTGG | CCT1202sgEF | CG14059 | CATCGGAGTCTGTTGCCTGATGG |
| CCT1092sgEF | CG13702 | CATACTGAATCTGGCTATCGCGG | CCT1203sgEF | CG14167 | CTACTAATCCTTATGATCGGCGG |
| CCT1092sgEF | CG13702 | TGTAGCTGTACGAACAGTCCGGG | CCT1203sgEF | CG14167 | GCGGCCGCACAACTTCATGGTGG |
| CCT1092sgEF | CG13702 | GTCCAGGTGGGGTCGCACACTGG | CCT1203sgEF | CG14167 | GTAGGAGTAGGCTAGGTAGCAGG |
| CCT1093sgEF | CG13968 | TTATCATAGAGATTGCTCAGGGG | CCT1204sgEF | CG14173 | CCCGGAAACCACAAACTCTGCGG |
| CCT1093sgEF | CG13968 | GATGCAGCAGCCCGCCCAGGCGG | CCT1204sgEF | CG14173 | TGCAGGATGATAGCAGCATGTGG |
| CCT1093sgEF | CG13968 | ACTGAGTCAAGGCTGCGCCTTGG | CCT1204sgEF | CG14173 | GCATGTGGCAGACACTGGACGGG |
| CCT1094sgEF | CG11937 | TACAACGCGTCGCCGCAACGCGG | CCT1205sgEF | CG14920 | AAATCTCCTCCGGCTGACTGGGG |
| CCT1094sgEF | CG11937 | TTCGGCTCTCTCTCTTCGGCTGG | CCT1205sgEF | CG14920 | AGAAGTTGCACAAGTCTTGCCGG |
| CCT1094sgEF | CG11937 | TTCGAAAGGTAGCGCAGCACTGG | CCT1205sgEF | CG14920 | AGCCGGAGAGGTGACATTGCCGG |
| CCT1096sgEF | CG13419 | GCTCCGCCACGAGAACAACAAGG | CCT1206sgEF | CG17673 | TCCAGGCCTGGGAATGGCCGTGG |
| CCT1096sgEF | CG13419 | TCAAATACCATTATCCGTGGCGG | CCT1206sgEF | CG17673 | TTTGTAGGCTTCCTATTCCACGG |
| CCT1096sgEF | CG13419 | CAGCATTCTGAAACTCTGCACGG | CCT1206sgEF | CG17673 | ACTCGGCTTGGTCCAGGCCTGGG |
| CCT1097sgEF | CG15520 | GACCACGACAAGAACCGACGAGG | CCT1207sgEF | CG17878 | CATTGGCCAACGCTCACCACTGG |
| CCT1097sgEF | CG15520 | GAAGGCATAGAGCCCCATGTTGG | CCT1207sgEF | CG17878 | CAGTTGAGAAGACCCCATCTCGG |
| CCT1097sgEF | CG15520 | ATCGCCGAATTCAGTACAGCTGG | CCT1207sgEF | CG17878 | ACTCAATGGGAAAGGACGCTTGG |
| CCT1098sgEF | CG4910 | CGTCCATGAGGATTTCCCTGAGG | CCT1208sgEF | CG33273 | GGAGAACTGGGATCACGGAGCGG |
| CCT1098sgEF | CG4910 | AGCTAAGTGGCGTTATACAATGG | CCT1208sgEF | CG33273 | TCAGCATGTCCATCAAGGCGGGG |
| CCT1098sgEF | CG4910 | GAACAACGAGGGCACCAATATGG | CCT1208sgEF | CG33273 | AGCTATCCAAATCCGCCAAGTGG |
| CCT1099sgEF | CG11318 | GTACGTTTCCACTCCAGTAGTGG | CCT1209sgEF | CG40041 | TATACGTGTATACACGACGATGG |
| CCT1099sgEF | CG11318 | TATGAAACCTGGTCAACTGAGGG | CCT1209sgEF | CG40041 | GTTGGGACTATGTAAGCGTTTGG |
| CCT1099sgEF | CG11318 | TGGAGAGGAACGTAATTCATCGG | CCT1209sgEF | CG40041 | CGCTGGGCGTGGACACAAACAGG |
| CCT1100sgEF | CG12290 | GCAGTCCCAGGAACAAAGTGGGG | CCT1210sgEF | CG6736 | GATTTACACGCCGTGTCAGGCGG |
| CCT1100sgEF | CG12290 | CTCCGTGGGTAAGCTCTCAAAGG | CCT1210sgEF | CG6736 | CGGCTGCAGTAACTGCGACACGG |
| CCT1100sgEF | CG12290 | GGGATCCGTAACGGCACACCAGG | CCT1210sgEF | CG6736 | GTGCGGCGAGGCTCTGATCCAGG |
| CCT1101sgEF | CG12796 | GATTGGAGATGACCGATCGCAGG | CCT1211sgEF | CG8167 | CTGCAGTGAAAAGCTCAACGAGG |
| CCT1101sgEF | CG12796 | GAGCTTCGATAATCCCAAGCGGG | CCT1211sgEF | CG8167 | GAAGGACAAAGGCTTGCTCATGG |
| CCT1101sgEF | CG12796 | CAGCAGGCAGGGACCTCTCATGG | CCT1211sgEF | CG8167 | GCGCGCTTGTGTGGAATCACGGG |
| CCT1102sgEF | CG13229 | TGTCGTTGAAAGGAACCCGCTGG | CCT1212sgEF | CG17777 | TATGGACACGGTCACTACGGCGG |
| CCT1102sgEF | CG13229 | AGATTTGAGCTACACCTGGGCGG | CCT1212sgEF | CG17777 | CGTCTTCTTCTCATCCACGGCGG |
| CCT1102sgEF | CG13229 | TTCCAGCATCACAAACATATCGG | CCT1212sgEF | CG17777 | CGCCGTAGCCGCCATAGCCGTGG |
| CCT1103sgEF | CG15556 | GGATCACGTGACCTGTAGGTCGG | CCT1213sgEF | CG45777 | CGTGTCTTGGGCGAGCACGGAGG |
| CCT1103sgEF | CG15556 | TATGAGACCTGGCTTAGCGATGG | CCT1213sgEF | CG45777 | GGAGGCGAGTGCCGTGCCCACGG |
| CCT1103sgEF | CG15556 | TGAGTCGGAACGATGTACTCTGG | CCT1213sgEF | CG45777 | GGAGCGTTGCTAAAGGAAGCGGG |
| CCT1104sgEF | CG15614 | GATGCAGTCCATCCACGAAAGGG | CCT1214sgEF | CG34136 | GAAGAATGCAAGGCACAGTAGGG |
| CCT1104sgEF | CG15614 | TCACCAGCTCAGAATCCGGTTGG | CCT1214sgEF | CG34136 | GAATGAGGTGAGCGGAAGACAGG |
| CCT1104sgEF | CG15614 | AAAGTTCGTACACAGTCCAAAGG | CCT1214sgEF | CG34136 | TTGCCCAAGCGAAGAATGCAAGG |
| CCT1105sgEF | CG15744 | GCTCTCCCTGGGCACACCAATGG | CCT1215sgEF | CG43117 | GAATTGGTCTGGGCTGAGGAAGG |
| CCT1105sgEF | CG15744 | GCCATTAGCCACATCTATGAGGG | CCT1215sgEF | CG43117 | ATCCACCAACACCACAACCACGG |
| CCT1105sgEF | CG15744 | CGCCTGGCCCCCCTCTGCTGCGG | CCT1215sgEF | CG43117 | GCAGGCAGAATCCGGCGAATAGG |
| CCT1107sgEF | CG30340 | CTGGCTGCACAAGCCCAATGGGG |  |  |  |
| CCT1107sgEF | CG30340 | GACTACCGAGAGATTGAGCACGG |  |  |  |
| CCT1107sgEF | CG30340 | CTTCTATCAGAACTACCAGTTGG |  |  |  |
