# Supplementary material for: Conditional chemoconnectomics (cCCTomics) as a strategy for efficient and conditional targeting of chemical transmission
Source: eLife. 2024 Apr 30;12:RP91927. doi: 10.7554/eLife.91927 (PMC11060718; doi:10.7554/eLife.91927)
Supplement: Supplementary file 4. [file elife-91927-supp4.docx]

**CCT gene expression profile of clock neuron**

|  | s-LNv | | l-LNv | | 5th | | LNd | | LPN | | DN1 | | DN2 | | DN3 | |
| --- | --- | --- | --- | --- | --- | --- | --- | --- | --- | --- | --- | --- | --- | --- | --- | --- |
| Gene symbol | GFP+ | N^#^ | GFP+ | N^#^ | GFP+ | N^#^ | GFP+ | N^#^ | GFP+ | N^#^ | GFP+ | N^#^ | GFP+ | N^#^ | GFP+ | N^#^ |
| AstC | / | / | / | / | / | / | 1 ± 0 | 6 | / | / | / | / | 1.83 ± 0.41 | 6 | *1 ± 0* | *1* |
| CCHa1 | / | / | / | / | / | / | / | / | / | / | **1.6 ± 0.55** | 5 | / | / | / | / |
| ChAT | *3 ± 1.41* | *2* | / | / | / | / | **2.3 ± 1.06** | 10 | *1 ± 1* | *1* | ***1 ± 0*** | *1* | 1 ± 0 | 10 | **1 ± 0** | 7 |
| CNMa | / | / | / | / | / | / | / | / | / | / | **4 ± 1.41** | 2 | / | / | 1 ± 0 | 2 |
| Dh31 | 3.64 ± 0.5 | 11 | / | / | / | / | 1.2 ± 0.42 | 10 | / | / | **3.8 ± 0.63** | 10 | / | / | / | / |
| Dh44 | / | / | 1.67 ± 0.58 | 3 | / | / | **3 ± 1** | 3 | / | / | **5 ± 0** | 3 | 1.67 ± 0.58 | 3 | **1.67 ± 0.58** | 3 |
| GlyT | 2.8 ± 1.64 | 5 | 3.88 ± 0.35 | 8 | 1 ± 0 | 3 | 1 ± 0 | 3 | / | / | **1.57 ± 0.79** | 7 | 1 ± 0 | 6 | 2 ± 0.93 | 8 |
| ITP | / | / | / | / | / | / | / | / | / | / | / | / | / | / | 1 ± 0 | 2 |
| NPF | 1 ± 0 | 2 | / | / | / | / | **1 ± 0** | 3 | / | / | / | / | / | / | / | / |
| Nplp1 | / | / | / | / | / | / | / | / | / | / | **5 ± 0** | 2 | / | / | **2 ± 0** | 2 |
| Proc | / | / | / | / | / | / | **1 ± 0** | 12 | / | / | / | / | / | / | / | / |
| sNPF | **4 ± 0** | 7 | / | / | / | / | **1.63 ± 0.52** | 8 | / | / | / | / | / | / | / | / |
| TH | 1.5 ± 0.71 | 2 | / | / | / | / | 1 ± 0 | 2 | / | / | 3.5 ± 0.71 | 2 | / | / | / | / |
| Trh | / | / | / | / | / | / | / | / | / | / | 1.75 ± 0.5 | 4 | / | / | / | / |
| Trissin | / | / | / | / | / | / | **1.82 ± 0.4** | 11 | / | / | / | / | / | / | / | / |
| VGlut | / | / | 1.2 ± 0.45 | 5 | / | / | 1.17 ± 0.41 | 6 | / | / | **3.5 ± 0.55** | 6 | *2 ± 0* | *1* | 2.2 ± 0.45 | 5 |
| CG13229 | / | / | **3.5 ± 0.55** | 6 | *1 ± 0* | *1* | **1.5 ± 0.55** | 6 | / | / | / | / | / | / | 1 ± 0 | 3 |
| CG32547 | / | / | **2.71 ± 0.95** | 7 | / | / | **1.86 ± 0.9** | 7 | / | / | **3.29 ± 1.8** | 7 | 1 ± 0 | 5 | / | / |
| CG43795 | / | / | / | / | / | / | / | / | / | / | / | / | 1 ± 0 | 6 | / | / |
| 5-HT2B | / | / | / | / | / | / | **1.62 ± 0.65** | 13 | / | / | / | / | *1 ± 0* | *1* | / | / |
| CCHa1-R | 1.5 ± 0.71 | 2 | / | / | / | / | / | / | / | / | / | / | / | / | / | / |
| CNMaR | / | / | / | / | / | / | / | / | / | / | / | / | / | / | 15.67 ± 0.58 | 3 |
| CrzR | / | / | / | / | / | / | / | / | / | / | **2 ± 0** | 2 | 1.5 ± 0.71 | 2 | **2.5 ± 0.71** | 2 |
| D2R | / | / | / | / | / | / | 1 ± 0 | 6 | / | / | / | / | / | / | / | / |
| Dh31-R | ***2 ± 0*** | *2* | **3.43 ± 0.53** | 7 | *1 ± 0* | *2* | / | / | / | / | **1 ± 0** | 3 | / | / | / | / |
| Dh44-R2 | / | / | / | / | / | / | 1 ± 0 | 6 | / | / | / | / | / | / | / | / |
| FMRFaR | / | / | / | / | / | / | / | / | / | / | / | / | 1 ± 0 | 6 | / | / |
| GABA-B-R2 | 3.43 ± 0.53 | 7 | 3.63 ± 0.52 | 8 | 1 ± 0 | 4 | 4.29 ± 1.7 | 7 | / | / | 3 ± 0 | 6 | *1 ± 0* | *1* | *1 ± 0* | *1* |
| GABA-B-R3 | / | / | / | / | / | / | 1 ± 0 | 4 | / | / | / | / | / | / | / | / |
| GluRIB | / | / | / | / | / | / | / | / | / | / | **1.56 ± 1.01** | 9 | 1.5 ± 0.58 | 4 | *1 ± 0* | *2* |
| mAChR-B | 1.33 ± 0.58 | 3 | 3.33 ± 0.87 | 9 | / | / | 1.5 ± 0.53 | 8 | / | / | 3.38 ± 2.33 | 8 | 1 ± 0 | 5 | / | / |
| MsR1 | / | / | **3.29 ± 0.49** | 7 | / | / | **1 ± 0** | 7 | *1 ± 1* | *1* | / | / | 2 ± 0 | 1 | / | / |
| nAChRα1 | 3 ± 0 | 2 | / | / | / | / | **5.6 ± 0.89** | 5 | 2 ± 1 | 3 | **12.4 ± 1.52** | 5 | / | / | 1 ± 0 | 2 |
| nAChRα2 | / | / | 3.8 ± 0.45 | 5 | *1 ± 0* | *1* | 2 ± 0.89 | 6 | / | / | 2.17 ± 1.17 | 6 | / | / | 2 ± 0.71 | 5 |
| nAChRβ2 | / | / | 3 ± 1 | 3 | / | / | / | / | / | / | 1 ± 0 | 2 | / | / | 1 ± 0 | 5 |
| Nmdar1 | *1 ± 0* | *2* | 3.64 ± 0.5 | 11 | / | / | 1.88 ± 0.35 | 8 | / | / | 1.5 ± 0.58 | 4 | 1.11 ± 0.33 | 9 | 1.4 ± 0.84 | 10 |
| NPFR | / | / | / | / | / | / | 1 ± 0 | 10 | / | / | / | / | / | / | **1.14 ± 0.38** | 7 |
| Oct-TyrR | / | / | / | / | / | / | / | / | / | / | / | / | / | / | 2 ± 0 | 2 |
| Octβ2R | / | / | / | / | / | / | / | / | / | / | **10.5 ± 0.71** | 2 | **1.5 ± 0.71** | 2 | 2.5 ± 0.71 | 2 |
| Pdfr | / | / | 1.14 ± 0.38 | 7 | / | / | ***1 ± 0*** | *2* | / | / | **2.29 ± 1.11** | 7 | 1 ± 0 | 6 | 1.33 ± 0.58 | 3 |
| PK2-R1 | / | / | / | / | / | / | **1.5 ± 0.71** | 2 | / | / | / | / | 1 ± 0 | 3 | / | / |
| SIFaR | / | / | **3.21 ± 0.43** | 14 | / | / | **2 ± 0.39** | 14 | / | / | **1 ± 0** | 5 | 1 ± 0 | 6 | **1.22 ± 0.44** | 9 |
| spab | / | / | / | / | / | / | 1 ± 0 | 4 | / | / | / | / | 1 ± 0 | 2 | / | / |
|  |  |  |  |  |  |  |  |  |  |  |  |  |  |  |  |  |

N^#^ represents GFP positive brain number. The largest N# number indicates total brains analyzed for certain CCT line.

/ represents no GFP found in corresponding clock neuron subset

The *italic* values indicate that the number of positive brains is smaller than the total number of brains analyzed

The **bolder** values (41/127) indicate that related genes were also reported in transcriptome analysis (Ma et al., 2021)
