# Supplementary material for: Conditional chemoconnectomics (cCCTomics) as a strategy for efficient and conditional targeting of chemical transmission
Source: eLife. 2024 Apr 30;12:RP91927. doi: 10.7554/eLife.91927 (PMC11060718; doi:10.7554/eLife.91927)
Supplement: Supplementary file 5. [file elife-91927-supp5.docx]

**List of CCT genes intersected with Clk856 drivers**

| **RaoLab Stock No.** | **CG No.** | **Gene Symbol** | **Driver** | **Intersection with Clk856** |
| --- | --- | --- | --- | --- |
| DKI0289 | CG42796 | 5-HT2B | LexA | Clk856-GAL4 |
| DKI0317 | CG14919 | AstC | LexA | Clk856-GAL4 |
| DKI0292 | CG43795 | CG43795 | LexA | Clk856-GAL4 |
| DKI0303 | CG13229 | CG13229 | LexA | Clk856-GAL4 |
| DKI0073 | CG13995 | CG13995 | LexA | Clk856-GAL4 |
| DKI0110 | CG12345 | ChAT | LexA | Clk856-GAL4 |
| DKI0254 | CG32547 | CG32547 | LexA | Clk856-GAL4 |
| DKI0121 | CG33517 | Dop2R | LexA | Clk856-GAL4 |
| DKI0309 | CG12370 | Dh44-R2 | LexA | Clk856-GAL4 |
| DKI0060 | CG13094 | Dh31 | LexA | Clk856-GAL4 |
| DKI0105 | CG32843 | Dh31-R | LexA | Clk856-GAL4 |
| WCKI1122 | CG2114 | FMRFaR | LexA | Clk856-GAL4 |
| DKI0305 | CG5549 | GlyT | LexA | Clk856-GAL4 |
| DKI0322 | CG6706 | GABA-B-R2 | LexA | Clk856-GAL4 |
| DKI0312 | CG3022 | GABA-B-R3 | LexA | Clk856-GAL4 |
| DKI0191 | CG43743 | GluRIB | LexA | Clk856-GAL4 |
| DKI0212 | CG6798 | nAChRβ2 | LexA | Clk856-GAL4 |
| DKI0221 | CG7918 | mAChR-B | LexA | Clk856-GAL4 |
| DKI0201 | CG6844 | nAChRα2 | LexA | Clk856-GAL4 |
| WCKI1173 | CG8985 | MsR1 | LexA | Clk856-GAL4 |
| WCKI1006 | CG1147 | NPFR | LexA | Clk856-GAL4 |
| WCKI1018 | CG7105 | Proc | LexA | Clk856-GAL4 |
| DKI0093 | CG2902 | Nmdar1 | LexA | Clk856-GAL4 |
| WCKI1089 | CG10823 | SIFaR | LexA | Clk856-GAL4 |
| WCKI1080 | CG8784 | PK2-R1 | LexA | Clk856-GAL4 |
| DKI0059 | CG13968 | sNPF | LexA | Clk856-GAL4 |
| DKI0082 | CG14871 | Trissin | LexA | Clk856-GAL4 |
| DKI0248 | CG9122 | Trh | LexA | Clk856-GAL4 |
| DKI0265 | CG9887 | VGlut | LexA | Clk856-GAL4 |
| DKI0165 | CG13586 | ITP | Gal4 | Clk856-p65AD |
| DKI0111 | CG10118 | TH | Gal4 | Clk856-p65AD |
| DKI0168 | CG3441 | Nplp1 | Gal4 | Clk856-p65AD |
| DKI0287 | CG14723 | HisCl1 | Gal4 | Clk856-p65AD |
| DKI0203 | CG5610 | nAChRα1 | Gal4 | Clk856-p65AD |
| WCKI1015 | CG10342 | NPF | LexA | Clk856-GAL4 |
| DKI0099 | CG14358 | CCHa1 | LexA | Clk856-GAL4 |
| WCKI1017 | CG33976 | Octβ2R | Gal4 | Clk856-p65AD |
| WCKI1019 | CG10698 | CrzR | Gal4 | Clk856-p65AD |
| DKI0078 | CG30106 | CCHa1-R | LexA | Clk856-GAL4 |
| DKI0029 | CG13936 | CNMa | p65AD | Clk856-GAL4 |
| DKI0288 | CG33696 | CNMaR | p65AD | Clk856-GAL4 |
| WCKI1095 | CG8348 | Dh44 | GAL4 | Clk856-p65AD |
| DKI0134 | CG13758 | Pdfr | LexA | Clk856-GAL4 |
| WCKI1199 | CG7485 | Oct-TyrR | Gal4 | Clk856-p65AD |
| DKI0262 | CG8216 | spab | LexA | Clk856-GAL4 |
| DKI0013 | CG10537 | Rdl | LexA | Clk856-GAL4 |
| DKI0069 | CG1056 | 5-HT2A | LexA | Clk856-GAL4 |
| WCKI1162 | CG10626 | Lkr | LexA | Clk856-GAL4 |
| DKI0251 | CG11318 | CG11318 | LexA | Clk856-GAL4 |
| WCKI1030 | CG11325 | AkhR | LexA | Clk856-GAL4 |
| WCKI1201 | CG1171 | Akh | LexA | Clk856-GAL4 |
| DKI0215 | CG11822 | nAChRβ3 | LexA | Clk856-GAL4 |
| CG11883-L-LexA | CG11883 | CG11883 | LexA | Clk856-GAL4 |
| CG11883-S-LexA | CG11883 | CG11883 | LexA | Clk856-GAL4 |
| WCKI1180 | CG11937 | amn | LexA | Clk856-GAL4 |
| DKI0307 | CG12344 | CG12344 | LexA | Clk856-GAL4 |
| Nplp3-LexA | CG13061 | Nplp3 | LexA | Clk856-GAL4 |
| L53-T5-W- | CG13480 | Lk | LexA | Clk856-GAL4 |
| DKI0302 | CG13565 | Orcokinin | LexA | Clk856-GAL4 |
| DKI0050 | CG13575 | CG13575 | LexA | Clk856-GAL4 |
| DKI0160 | CG13579 | CG13579 | LexA | Clk856-GAL4 |
| DKI0286 | CG13579 | CG13579-RB | LexA | Clk856-GAL4 |
| DKI0091 | CG13633 | AstA | LexA | Clk856-GAL4 |
| DKI0096 | CG14375 | CCHa2 | LexA | Clk856-GAL4 |
| DKI0086 | CG14575 | CapaR | LexA | Clk856-GAL4 |
| DKI0007 | CG14593 | CCHa2-R | LexA | Clk856-GAL4 |
| DKI0094 | CG14734 | Tk | LexA | Clk856-GAL4 |
| DKI0020 | CG14994 | gad1 | LexA | Clk856-GAL4 |
| DKI0089 | CG15113 | 5-HT1B | LexA | Clk856-GAL4 |
| DKI0187 | CG15274 | GABA-B-R1 | LexA | Clk856-GAL4 |
| DKI0055 | CG15284 | Pburs | LexA | Clk856-GAL4 |
| Tβh-LexA | CG1543 | Tβh | LexA | Clk856-GAL4 |
| DKI0143 | CG15520 | Capa | LexA | Clk856-GAL4 |
| WCKI1196 | CG15614 | CG15614 | LexA | Clk856-GAL4 |
| DKI0159 | CG15744 | CG15744 | LexA | Clk856-GAL4 |
| DKI0030 | CG16720 | 5-HT1A | LexA | Clk856-GAL4 |
| DKI0146 | CG16752 | SPR | LexA | Clk856-GAL4 |
| DKI0156 | CG16992 | mthl6 | LexA | Clk856-GAL4 |
| DKI0294 | CG17061 | mthl10 | LexA | Clk856-GAL4 |
| WCKI1120 | CG17084 | CG17084 | LexA | Clk856-GAL4 |
| DKI0031 | CG17795 | mthl2 | LexA | Clk856-GAL4 |
| DKI0196 | CG18039 | GluRIID | LexA | Clk856-GAL4 |
| DKI0148 | CG18090 | Dsk | LexA | Clk856-GAL4 |
| WCKI1097 | CG18208 | CG18208 | LexA | Clk856-GAL4 |
| DKI0321 | CG18314 | DopEcR | LexA | Clk856-GAL4 |
| DKI0123 | CG18741 | DopR2 | LexA | Clk856-GAL4 |
| DKI0205 | CG2302 | nAChR α3 | LexA | Clk856-GAL4 |
| WCKI1184 | CG2346 | FMRFa | LexA | Clk856-GAL4 |
| DKI0225 | CG2872 | AstA-R1 | LexA | Clk856-GAL4 |
| DKI0290 | CG30018 | mthl13 | LexA | Clk856-GAL4 |
| DKI0066 | CG30340 | CG30340 | LexA | Clk856-GAL4 |
| DKI0151 | CG31096 | Lgr3 | LexA | Clk856-GAL4 |
| DKI0231 | CG31147 | mthl11 | LexA | Clk856-GAL4 |
| DKI0232 | CG31760 | CG31760 | LexA | Clk856-GAL4 |
| DKI0234 | CG32447 | CG32447 | LexA | Clk856-GAL4 |
| DKI0259 | CG32475 | mthl8 | LexA | Clk856-GAL4 |
| WCKI1107 | CG32476 | CG32476 | LexA | Clk856-GAL4 |
| DKI0252 | CG32540 | CCKLR-17D3 | LexA | Clk856-GAL4 |
| DKI0206 | CG32975 | nAChR α5 | LexA | Clk856-GAL4 |
| DKI0101 | CG33344 | CCAP-R | LexA | Clk856-GAL4 |
| DKI0284 | CG33495 | Dup99B | LexA | Clk856-GAL4 |
| DKI0087 | CG33495 | Dup99B | LexA | Clk856-GAL4 |
| DKI0128 | CG33513 | Nmdar2 | LexA | Clk856-GAL4 |
| DKI0315 | CG33527 | SIFa | LexA | Clk856-GAL4 |
| DKI0180 | CG33639 | CG33639 | LexA | Clk856-GAL4 |
| DKI0246 | CG34388 | natalisin | LexA | Clk856-GAL4 |
| DKI0175 | CG34411 | CG34411 | LexA | Clk856-GAL4 |
| DKI0102 | CG3454 | HDC | LexA | Clk856-GAL4 |
| WCKI1101 | CG3856 | oamb | LexA | Clk856-GAL4 |
| DKI0208 | CG4128 | nAChR α6 | LexA | Clk856-GAL4 |
| WCKI1044 | CG42244 | Octβ3R | LexA | Clk856-GAL4 |
| DKI0193 | CG4226 | GluRIIC | LexA | Clk856-GAL4 |
| DKI0173 | CG42301 | CCKLR-17D1 | LexA | Clk856-GAL4 |
| WCKI1166 | CG4313 | CG4313 | LexA | Clk856-GAL4 |
| DKI0276 | CG4356 | mAChR-A | LexA | Clk856-GAL4 |
| DKI0016 | CG43745 | MsR2 | LexA | Clk856-GAL4 |
| WCKI1169 | CG4395 | hec | LexA | Clk856-GAL4 |
| NT5E1-lexA | CG4827 | NT5E-1 | LexA | Clk856-GAL4 |
| WCKI1176 | CG5400 | Eh | LexA | Clk856-GAL4 |
| WCKI1203 | CG5811 | RYa-R | LexA | Clk856-GAL4 |
| WCKI1205 | CG5911 | ETHR | LexA | Clk856-GAL4 |
| WCKI1055 | CG6371 | CG6371 | LexA | Clk856-GAL4 |
| WCKI1009 | CG6440 | Ms | LexA | Clk856-GAL4 |
| WCKI1011 | CG6456 | Mip | LexA | Clk856-GAL4 |
| WCKI1192 | CG6530 | mthl3 | LexA | Clk856-GAL4 |
| WCKI1194 | CG6536 | mthl4 | LexA | Clk856-GAL4 |
| WCKI1049 | CG6919 | oa2 | LexA | Clk856-GAL4 |
| WCKI1118 | CG6965 | CG6965 | LexA | Clk856-GAL4 |
| DKI0192 | CG6992 | GluRIIA | LexA | Clk856-GAL4 |
| WCKI1093 | CG7285 | AstC-R1 | LexA | Clk856-GAL4 |
| DKI0071 | CG7411 | Ort | LexA | Clk856-GAL4 |
| DKI0311 | CG7431 | TyrR | LexA | Clk856-GAL4 |
| DKI0299 | CG7446 | Grd | LexA | Clk856-GAL4 |
| DKI0320 | CG7497 | CG7497 | LexA | Clk856-GAL4 |
| WCKI1094 | CG7665 | Lgr1 | LexA | Clk856-GAL4 |
| DKI0240 | CG8380 | DAT | LexA | Clk856-GAL4 |
| DKI0047 | CG8394 | vGAT | LexA | Clk856-GAL4 |
| WCKI1181 | CG8422 | Dh44-R1 | LexA | Clk856-GAL4 |
| DKI0269 | CG8442 | GluRIA | LexA | Clk856-GAL4 |
| WCKI1174 | CG8795 | PK2-R2 | LexA | Clk856-GAL4 |
| WCKI1060 | CG10001 | CG10001 | Gal4 | Clk856-p65AD |
| DKI0130 | CG14723 | HisCl1 | Gal4 | Clk856-p65AD |
| DKI0041 | CG15361 | Nplp4 | Gal4 | Clk856-p65AD |
| WCKI1198 | CG15556 | CG15556 | Gal4 | Clk856-p65AD |
| DKI0255 | CG18105 | ETH | Gal4 | Clk856-p65AD |
| DKI0198 | CG31201 | GluRIIE | Gal4 | Clk856-p65AD |
| WCKI1164 | CG3171 | Tre1 | Gal4 | Clk856-p65AD |
| DKI0300 | CG4910 | CCAP | Gal4 | Clk856-p65AD |
| DKI0226 | CG6936 | mth | Gal4 | Clk856-p65AD |
| WCKI1178 | CG6986 | Proc-R | Gal4 | Clk856-p65AD |
| DKI0297 | CG7476 | mthl7 | Gal4 | Clk856-p65AD |
| WCKI1053 | CG9918 | PK1-R | Gal4 | Clk856-p65AD |
