# Supplementary material for: Conditional chemoconnectomics (cCCTomics) as a strategy for efficient and conditional targeting of chemical transmission
Source: eLife. 2024 Apr 30;12:RP91927. doi: 10.7554/eLife.91927 (PMC11060718; doi:10.7554/eLife.91927)
Supplement: Supplementary file 6. [file elife-91927-supp6.docx]

**Phenotypes of CCT genes knocking out in clock neurons**

| **Target Gene** | **LD condition** | | | | **DD conditon** | | |
| --- | --- | --- | --- | --- | --- | --- | --- |
|  | **MAI** | **MAPI** | **EAI** | **EAPI** | **Power** | **Period** | **AR** |
| **Pdfr** | **0.087 ± 0.119** | -0.047 ± 0.175 | 0.34 ± 0.076 | **0.101 ± 0.071** | **3.45 ± 7.54** | **22.41 ± 0.51** | 17/22 |
| **Pdf** | **0.11 ± 0.124** | 0.026 ± 0.12 | 0.383 ± 0.065 | **0.114 ± 0.094** | **15.46 ± 34.25** | **22.31 ± 0.4** | 13/23 |
| **nAChRα1** | 0.316 ± 0.121 | **0.14 ± 0.145** | 0.352 ± 0.094 | -0.094 ± 0.068 | **37.83 ± 27.68** | **23.18 ± 0.43** | 1/14 |
| **Dh44** | 0.339 ± 0.111 | 0.042 ± 0.106 | 0.367 ± 0.09 | -0.138 ± 0.088 | **44.22 ± 45.84** | 23.55 ± 0.24 | 7/19 |
| **CNMaR** | 0.345 ± 0.063 | 0.062 ± 0.129 | 0.342 ± 0.132 | -0.042 ± 0.061 | **71.37 ± 39.43** | 24.01 ± 0.8 | 1/22 |
| **SIFaR** | 0.296 ± 0.076 | 0.062 ± 0.098 | 0.381 ± 0.08 | -0.041 ± 0.078 | **75.56 ± 66.38** | 23.32 ± 0.33 | 4/24 |
| ITP | 0.277 ± 0.103 | 0.015 ± 0.106 | 0.357 ± 0.058 | -0.06 ± 0.075 | 80.74 ± 39.06 | 23.75 ± 0.29 | 2/24 |
| CG17777 | 0.272 ± 0.096 | -0.025 ± 0.18 | 0.453 ± 0.068 | -0.114 ± 0.048 | 81.81 ± 49.34 | 23.74 ± 0.29 | 1/22 |
| **Dh31** | **0.205 ± 0.122** | -0.035 ± 0.134 | 0.405 ± 0.075 | -0.092 ± 0.094 | 82.44 ± 51.16 | 23.5 ± 0.29 | 1/22 |
| CG13995 | 0.346 ± 0.087 | 0.011 ± 0.152 | 0.414 ± 0.055 | -0.072 ± 0.099 | 87.7 ± 36.53 | 23.56 ± 0.33 | 1/24 |
| nAChRα2 | 0.26 ± 0.1 | 0 ± 0.145 | 0.348 ± 0.088 | -0.042 ± 0.078 | 88.15 ± 50.2 | 23.7 ± 0.24 | 1/22 |
| NPFR | 0.269 ± 0.174 | 0.042 ± 0.092 | 0.403 ± 0.089 | -0.072 ± 0.073 | 88.69 ± 46.62 | 23.35 ± 0.33 | 2/24 |
| CrzR | 0.324 ± 0.087 | 0.026 ± 0.122 | 0.415 ± 0.068 | -0.028 ± 0.083 | 91.63 ± 54.11 | 23.35 ± 0.39 | 2/24 |
| HisCl1 | 0.306 ± 0.075 | 0.08 ± 0.097 | 0.398 ± 0.04 | -0.04 ± 0.096 | 93.7 ± 48.07 | 23.8 ± 0.39 | 1/23 |
| AstC-R2 | 0.363 ± 0.074 | -0.073 ± 0.164 | 0.465 ± 0.082 | -0.136 ± 0.073 | 94.45 ± 57.72 | 24.04 ± 0.38 | 1/24 |
| VGAT | 0.249 ± 0.093 | 0.078 ± 0.118 | 0.402 ± 0.054 | -0.106 ± 0.078 | 98.11 ± 52.33 | 23.42 ± 0.36 | 1/21 |
| MsR1 | 0.31 ± 0.069 | 0.091 ± 0.089 | 0.336 ± 0.086 | -0.099 ± 0.077 | 99.46 ± 46.48 | 24.14 ± 0.65 | 1/24 |
| CCAP | 0.321 ± 0.075 | -0.028 ± 0.191 | 0.464 ± 0.06 | -0.105 ± 0.074 | 99.76 ± 57.98 | 23.84 ± 0.37 | 0/22 |
| CG34136 | 0.278 ± 0.084 | 0.022 ± 0.099 | 0.388 ± 0.052 | -0.037 ± 0.085 | 101.43 ± 54.01 | 23.7 ± 0.43 | 1/22 |
| GABA-B-R2 | 0.277 ± 0.078 | -0.016 ± 0.108 | 0.337 ± 0.074 | -0.084 ± 0.071 | 101.92 ± 30.51 | 23.95 ± 0.4 | 0/20 |
| Hug | 0.337 ± 0.073 | 0.013 ± 0.093 | 0.41 ± 0.042 | -0.059 ± 0.081 | 102 ± 51.47 | 23.55 ± 0.39 | 1/23 |
| **nAChRβ2** | 0.254 ± 0.095 | 0.035 ± 0.121 | 0.355 ± 0.079 | **-0.011 ± 0.07** | 104.34 ± 42.52 | 23.91 ± 0.3 | 0/22 |
| AstC | 0.329 ± 0.102 | -0.005 ± 0.178 | 0.36 ± 0.098 | -0.017 ± 0.057 | 104.4 ± 48.76 | 23.71 ± 0.4 | 2/23 |
| Nplp3 | 0.38 ± 0.06 | 0.023 ± 0.102 | 0.415 ± 0.041 | -0.124 ± 0.076 | 105.02 ± 47.1 | 23.66 ± 0.35 | 1/22 |
| Ptth | 0.377 ± 0.069 | -0.028 ± 0.127 | 0.409 ± 0.057 | -0.061 ± 0.085 | 105.4 ± 35.61 | 23.49 ± 0.32 | 0/24 |
| GluRIB | 0.317 ± 0.079 | -0.016 ± 0.137 | 0.363 ± 0.057 | -0.059 ± 0.077 | 106.25 ± 41.48 | 23.78 ± 0.25 | 0/18 |
| CCHa1-R | 0.307 ± 0.1 | 0.052 ± 0.088 | 0.419 ± 0.041 | -0.064 ± 0.073 | 106.83 ± 46.29 | **23.28 ± 0.4** | 0/23 |
| **TβH** | 0.257 ± 0.103 | 0.037 ± 0.123 | **0.31 ± 0.061** | -0.05 ± 0.079 | 107.86 ± 44.06 | 23.74 ± 0.38 | 0/19 |
| Oct-TyrR | 0.343 ± 0.085 | 0.013 ± 0.107 | 0.387 ± 0.061 | -0.088 ± 0.1 | 108.19 ± 56.73 | 23.94 ± 0.32 | 2/22 |
| ***Clk856-GAL4>Cas9.M9*** | ***0.3 ± 0.076*** | ***0.016 ± 0.09*** | ***0.38 ± 0.048*** | ***-0.066 ± 0.074*** | ***108.37 ± 30.19*** | ***23.8 ± 0.25*** | ***0/24*** |
| Dop2R | 0.357 ± 0.075 | 0.063 ± 0.137 | 0.425 ± 0.084 | -0.076 ± 0.073 | 109.27 ± 69.88 | 23.51 ± 0.51 | 2/24 |
| Mip | 0.316 ± 0.052 | 0 ± 0.134 | 0.402 ± 0.048 | -0.068 ± 0.066 | 111.37 ± 42.75 | 23.47 ± 0.42 | 1/22 |
| TrH | 0.26 ± 0.124 | 0.05 ± 0.124 | 0.365 ± 0.051 | -0.024 ± 0.07 | 111.5 ± 41 | 23.7 ± 0.44 | 0/23 |
| Trissin | 0.261 ± 0.086 | 0.056 ± 0.116 | 0.362 ± 0.065 | -0.073 ± 0.056 | 111.89 ± 54.79 | 23.4 ± 0.29 | 2/22 |
| CG43795 | 0.333 ± 0.113 | 0.007 ± 0.114 | 0.415 ± 0.054 | -0.099 ± 0.071 | 111.9 ± 57.29 | 23.57 ± 0.35 | 0/22 |
| CG7589 | 0.327 ± 0.061 | 0.013 ± 0.12 | 0.474 ± 0.065 | -0.092 ± 0.07 | 112.13 ± 45.66 | 23.8 ± 0.38 | 0/23 |
| CNMa | 0.284 ± 0.086 | 0.082 ± 0.12 | 0.364 ± 0.041 | -0.091 ± 0.085 | 112.6 ± 41.9 | 23.97 ± 0.22 | 0/24 |
| CG32547 | 0.249 ± 0.098 | -0.015 ± 0.116 | 0.344 ± 0.078 | -0.049 ± 0.072 | 112.98 ± 63.4 | 23.69 ± 0.29 | 2/23 |
| CG45777 | 0.36 ± 0.067 | -0.047 ± 0.154 | 0.484 ± 0.065 | -0.119 ± 0.077 | 113.47 ± 68.45 | 23.55 ± 0.25 | 0/24 |
| Octβ2R | 0.319 ± 0.081 | 0.028 ± 0.065 | 0.434 ± 0.07 | -0.061 ± 0.071 | 114.28 ± 63.82 | 23.55 ± 0.41 | 1/24 |
| Dh44-R2 | 0.357 ± 0.058 | 0.054 ± 0.067 | 0.403 ± 0.047 | -0.039 ± 0.07 | 115.91 ± 61.1 | 23.51 ± 0.39 | 1/23 |
| VAChT | 0.284 ± 0.095 | -0.118 ± 0.116 | 0.401 ± 0.084 | -0.154 ± 0.058 | 116.69 ± 74.92 | 23.93 ± 0.3 | 1/21 |
| CCHa1 | 0.352 ± 0.058 | 0.002 ± 0.086 | 0.39 ± 0.062 | -0.119 ± 0.081 | 116.75 ± 44.06 | 23.73 ± 0.32 | 1/22 |
| mGluRA | 0.321 ± 0.065 | -0.005 ± 0.078 | 0.49 ± 0.047 | -0.062 ± 0.073 | 116.82 ± 50.26 | 24.04 ± 0.25 | 1/23 |
| PK2-R1 | 0.333 ± 0.079 | 0.037 ± 0.121 | 0.429 ± 0.046 | -0.099 ± 0.093 | 117.33 ± 50.14 | 23.95 ± 0.27 | 1/22 |
| **spab** | 0.253 ± 0.08 | -0.005 ± 0.094 | 0.363 ± 0.046 | **0.002 ± 0.057** | 117.38 ± 46.52 | 23.64 ± 0.26 | 0/22 |
| Dh31-R | 0.321 ± 0.103 | -0.021 ± 0.161 | 0.412 ± 0.043 | -0.09 ± 0.072 | 118.71 ± 50.62 | 23.6 ± 0.23 | 1/20 |
| FMRFaR | 0.294 ± 0.092 | -0.013 ± 0.099 | 0.373 ± 0.033 | -0.111 ± 0.074 | 119.47 ± 42.54 | 23.86 ± 0.32 | 0/21 |
| CG13229 | 0.359 ± 0.069 | 0.059 ± 0.098 | 0.421 ± 0.049 | -0.068 ± 0.066 | 122.46 ± 60.2 | 23.36 ± 0.37 | 2/21 |
| mAChR-B | 0.242 ± 0.112 | 0.09 ± 0.087 | 0.388 ± 0.037 | -0.047 ± 0.081 | 122.88 ± 40.64 | 23.77 ± 0.42 | 0/23 |
| 5-HT2B | 0.293 ± 0.085 | 0.035 ± 0.123 | 0.373 ± 0.042 | -0.095 ± 0.084 | 124.65 ± 34.31 | 24.03 ± 0.29 | 0/24 |
| TH | 0.3 ± 0.101 | 0.016 ± 0.131 | 0.349 ± 0.11 | -0.032 ± 0.086 | 125.71 ± 44.74 | 23.94 ± 0.33 | 1/23 |
| Proc | 0.362 ± 0.072 | 0.028 ± 0.142 | 0.437 ± 0.03 | -0.086 ± 0.095 | 128.13 ± 61.01 | 23.57 ± 0.37 | 0/23 |
| AstA | 0.312 ± 0.092 | -0.025 ± 0.113 | 0.412 ± 0.051 | -0.082 ± 0.089 | 131.22 ± 35.09 | 23.63 ± 0.31 | 0/24 |
| 5-HT1B | 0.313 ± 0.072 | 0.047 ± 0.117 | 0.402 ± 0.062 | -0.083 ± 0.091 | 132.29 ± 41.37 | 23.5 ± 0.29 | 0/20 |
| sNPF-R | 0.283 ± 0.112 | 0.035 ± 0.069 | 0.387 ± 0.035 | -0.089 ± 0.056 | 133.38 ± 41.77 | 23.74 ± 0.4 | 0/21 |
| CG43117 | 0.285 ± 0.084 | -0.03 ± 0.134 | 0.333 ± 0.081 | -0.095 ± 0.092 | 134.98 ± 58.52 | 23.72 ± 0.33 | 0/21 |
| NMDAR1 | 0.341 ± 0.066 | 0.065 ± 0.134 | 0.408 ± 0.052 | -0.084 ± 0.083 | 135.01 ± 45.81 | 23.76 ± 0.23 | 1/19 |
| GABA-B-R3 | 0.314 ± 0.104 | 0.041 ± 0.097 | 0.362 ± 0.061 | -0.081 ± 0.056 | 135.49 ± 37.61 | 23.79 ± 0.24 | 0/23 |
| CG12344 | 0.327 ± 0.062 | -0.043 ± 0.154 | 0.465 ± 0.099 | -0.121 ± 0.067 | 138.31 ± 63.27 | 23.98 ± 0.35 | 0/23 |
| Gpb5 | 0.268 ± 0.058 | -0.006 ± 0.12 | 0.424 ± 0.077 | -0.078 ± 0.068 | 138.41 ± 42.42 | 23.69 ± 0.29 | 0/23 |
| Nplp1 | 0.248 ± 0.115 | 0.072 ± 0.087 | 0.414 ± 0.023 | -0.071 ± 0.103 | 138.54 ± 40.39 | 23.92 ± 0.3 | 0/23 |
| Grd | 0.26 ± 0.091 | 0.008 ± 0.132 | 0.448 ± 0.075 | -0.065 ± 0.064 | 139.49 ± 78.28 | 23.65 ± 0.39 | 1/24 |
| NPF | 0.329 ± 0.086 | 0.007 ± 0.147 | 0.404 ± 0.067 | -0.071 ± 0.05 | 139.57 ± 44.27 | 23.72 ± 0.3 | 0/23 |
| sNPF | 0.346 ± 0.084 | -0.007 ± 0.118 | 0.414 ± 0.062 | -0.111 ± 0.065 | 146.16 ± 58.23 | 23.6 ± 0.45 | 0/17 |
| GlyT | 0.273 ± 0.111 | -0.119 ± 0.117 | 0.395 ± 0.076 | -0.193 ± 0.105 | 105.325 ± 60.615 | 23.548 ± 0.245 | 5/43 |

1. VGlut and ChAT presented in Supplementary file 8

2. Genotype for each experimental group is Clk856-GAL4/UAS-sgRNA^TargetGene^; UAS-Cas9.M9/+. Genotype for control group is Clk856-GAL4/+; UAS-Cas9.M9/+

3. Value in **bolder** indicate they are outside of one standard deviation (SD) compared to control group
