# Supplementary material for: Conditional chemoconnectomics (cCCTomics) as a strategy for efficient and conditional targeting of chemical transmission
Source: eLife. 2024 Apr 30;12:RP91927. doi: 10.7554/eLife.91927 (PMC11060718; doi:10.7554/eLife.91927)
Supplement: Supplementary file 7. [file elife-91927-supp7.docx]

**arrhythmicity related to Figure 6R**

| Genotype | arrhythmicity | Arrhythmic rate |
| --- | --- | --- |
| +;UAS-Pdfr/+ | 2/44 | 4.55% |
| Pdfr-attpKO;UAS-Pdfr/+ | 29/44 | 65.91% |
| Pdfr-attpKO;;R79A11-GAL4/+ | 38/47 | 80.85% |
| Pdfr-attpKO;UAS-Pdfr/+; R79A11-GAL4/+ | 5/40 | 12.50% |
| Pdfr-attpKO;;R91F02-GAL4/+ | 36/48 | 75.00% |
| Pdfr-attpKO;UAS-Pdfr/+;R91F02-GAL4/+ | 9/40 | 22.50% |
| Pdfr-attpKO;;R51H05-GAL4/+ | 36/46 | 78.26% |
| Pdfr-attpKO;UAS-Pdfr/+;R51H05-GAL4/+ | 20/43 | 46.51% |
