# Supplementary material for: Conditional chemoconnectomics (cCCTomics) as a strategy for efficient and conditional targeting of chemical transmission
Source: eLife. 2024 Apr 30;12:RP91927. doi: 10.7554/eLife.91927 (PMC11060718; doi:10.7554/eLife.91927)
Supplement: Supplementary file 8. [file elife-91927-supp8.docx]

**Phenotypes of candidate CCT genes knockout in clock neurons**

| **Genotype** | **LD condition** | | | | **DD condition** | | |
| --- | --- | --- | --- | --- | --- | --- | --- |
|  | **MAI** | **MAPI** | **EAI** | **EAPI** | **Power** | **Period** | **AR** |
| sgRNA^Dh44^, Cas9.M6 | 0.329 ± 0.086 | -0.058 ± 0.119 | 0.38 ± 0.058 | -0.081 ± 0.065 | 66.38 ± 40.4 | 23.18 ± 0.31 | 2/22 |
| Clk856> sgRNA^Dh44^ | 0.354 ± 0.093 | 0.03 ± 0.131 | 0.444 ± 0.045 | -0.085 ± 0.066 | 94 ± 45.35 | 23.61 ± 0.34 | 2/20 |
| Clk856> sgRNA^Dh44^, Cas9.M6 | 0.346 ± 0.096 | -0.02 ± 0.165 | 0.397 ± 0.059 | -0.085 ± 0.077 | 74.64 ± 27.25 | 23.75 ± 0.31 | 0/20 |
| sgRNA^nAChRα1^, Cas9.M6 | 0.29 ± 0.089 | -0.064 ± 0.139 | 0.363 ± 0.056 | -0.071 ± 0.064 | 58.35 ± 43.82 | 23.53 ± 0.45 | 2/13 |
| Clk856> sgRNA^nAChRα1^ | 0.318 ± 0.096 | 0.019 ± 0.137 | 0.401 ± 0.052 | -0.08 ± 0.071 | 56.89 ± 45.03 | 23.66 ± 0.43 | 1/12 |
| Clk856> sgRNA^nAChRα1^, Cas9.M6 | 0.307 ± 0.154 | -0.031 ± 0.099 | 0.381 ± 0.056 | -0.074 ± 0.074 | 69.21 ± 52.1 | 23.71 ± 0.29 | 2/13 |
| sgRNA^ChAT^, Cas9.M6 | 0.292 ± 0.096 | 0.026 ± 0.155 | 0.291 ± 0.092 | -0.1 ± 0.055 | 91.48 ± 55.18 | 23.43 ± 0.41 | 3/19 |
| Clk856> sgRNA^ChAT^ | 0.298 ± 0.098 | -0.016 ± 0.157 | 0.353 ± 0.071 | -0.079 ± 0.051 | 78.66 ± 41.65 | 23.54 ± 0.25 | 1/22 |
| Clk856> sgRNA^ChAT^, Cas9.M6 | 0.22 ± 0.137 | 0.003 ± 0.123 | 0.318 ± 0.078 | -0.124 ± 0.075 | 49.35 ± 42.11 | 23.7 ± 0.2 | 4/14 |
| sgRNA^CNMa^, Cas9.M6 | 0.342 ± 0.076 | -0.141 ± 0.138 | 0.35 ± 0.091 | -0.088 ± 0.079 | 92.81 ± 39.89 | 23.98 ± 0.44 | 1/23 |
| Clk856> sgRNA^CNMa^ | 0.355 ± 0.181 | 0.019 ± 0.093 | 0.431 ± 0.05 | -0.121 ± 0.079 | 18.96 ± 22.19 | 23.8 ± 0.27 | 7/14 |
| Clk856> sgRNA^CNMa^, Cas9.M6 | 0.354 ± 0.11 | 0.113 ± 0.169 | 0.353 ± 0.086 | -0.072 ± 0.066 | 93.51 ± 48.39 | 24.17 ± 0.42 | 1/20 |
| sgRNA^VGlut^, Cas9.M6 | 0.254 ± 0.114 | -0.115 ± 0.14 | 0.32 ± 0.066 | -0.073 ± 0.043 | 56.28 ± 39.85 | 23.41 ± 0.38 | 1/20 |
| Clk856> sgRNA^VGlut^ | 0.306 ± 0.102 | -0.026 ± 0.146 | 0.369 ± 0.074 | -0.104 ± 0.067 | 63.82 ± 48.6 | 23.6 ± 0.36 | 5/20 |
| Clk856> sgRNA^VGlut^, Cas9.M6 | 0.179 ± 0.1** | -0.087 ± 0.107 | 0.27 ± 0.07 | -0.158 ± 0.063 | 45.09 ± 41.35 | 23.7 ± 0.27 | 6/21 |
| sgRNA^mAChR-B^, Cas9.M6 | 0.37 ± 0.062 | -0.047 ± 0.109 | 0.374 ± 0.064 | -0.076 ± 0.054 | 110.16 ± 32.99 | 23.78 ± 0.47 | 0/21 |
| Clk856> sgRNA^mAChR-B^ | 0.358 ± 0.095 | -0.003 ± 0.109 | 0.442 ± 0.038 | -0.143 ± 0.087 | 73 ± 36 | 23.48 ± 0.45 | 0/17 |
| Clk856> sgRNA^mAChR-B^, Cas9.M6 | 0.259 ± 0.1 | -0.109 ± 0.139 | 0.357 ± 0.066 | -0.088 ± 0.074 | 109.35 ± 45.07 | 23.71 ± 0.36 | 0/22 |
| sgRNA^MsR1^, Cas9.M6 | 0.339 ± 0.078 | -0.011 ± 0.175 | 0.368 ± 0.061 | -0.072 ± 0.087 | 55.68 ± 49.3 | 23.77 ± 0.55 | 5/22 |
| Clk856> sgRNA^MsR1^ | 0.378 ± 0.098 | -0.023 ± 0.114 | 0.4 ± 0.036 | -0.143 ± 0.075 | 55.05 ± 43.19 | 23.89 ± 0.4 | 3/19 |
| Clk856> sgRNA^MsR1^, Cas9.M6 | 0.276 ± 0.085 | -0.04 ± 0.123 | 0.313 ± 0.063 | -0.085 ± 0.083 | 78.21 ± 50.06 | 23.85 ± 0.48 | 2/21 |
| sgRNA^SIFaR^, Cas9.M6 | 0.254 ± 0.104 | -0.075 ± 0.14 | 0.34 ± 0.047 | -0.072 ± 0.052 | 82.85 ± 42.29 | 23.63 ± 0.35 | 0/23 |
| Clk856> sgRNA^SIFaR^ | 0.329 ± 0.122 | -0.058 ± 0.121 | 0.375 ± 0.059 | -0.096 ± 0.05 | 81.71 ± 41.2 | 23.82 ± 0.36 | 0/21 |
| Clk856> sgRNA^SIFaR^, Cas9.M6 | 0.219 ± 0.143 | -0.142 ± 0.172 | 0.299 ± 0.095 | -0.117 ± 0.088 | 94.45 ± 32.78 | 24.04 ± 0.38 | 0/19 |
| Clk856> Cas9.M6 | 0.295 ± 0.12 | -0.043 ± 0.109 | 0.325 ± 0.081 | -0.118 ± 0.048 | 90.64 ± 43.86 | 23.7 ± 0.27 | 1/16 |

Clk856> sgRNA^VGlut^, Cas9.M6 vs Clk856> sgRNA^VGlut^ , ** *P＜0.01*

Clk856> sgRNA^VGlut^, Cas9.M6 vs Clk856> Cas9.M6 , ** *P＜0.01*

Clk856> sgRNA^VGlut^, Cas9.M6 vs sgRNA^VGlut^, Cas9.M6, *P = 0.0921*
