# Supplementary material for: Conditional chemoconnectomics (cCCTomics) as a strategy for efficient and conditional targeting of chemical transmission
Source: eLife. 2024 Apr 30;12:RP91927. doi: 10.7554/eLife.91927 (PMC11060718; doi:10.7554/eLife.91927)
Supplement: Supplementary file 9. [file elife-91927-supp9.docx]

**Conditional knockout of VGlut in DN1s**

| **Genotype** | **LD condition** | | | | **DD condition** | | |
| --- | --- | --- | --- | --- | --- | --- | --- |
|  | **MAI** | **MAPI** | **EAI** | **EAPI** | **POWER** | **PERIOD** | **AR** |
| sgRNA^VGlut^,Cas9.M6 | 0.268 ± 0.124 | -0.072 ± 0.106 | 0.328 ± 0.071 | -0.065 ± 0.035 | 79.06 ± 49.45 | 22.89 ± 0.19 | 1/23 |
| R18H11>sgRNA^VGlut^ | 0.258 ± 0.085 | -0.093 ± 0.104 | 0.327 ± 0.05 | -0.124 ± 0.069 | 67.49 ± 42.52 | 23.38 ± 0.36 | 1/21 |
| R18H11>sgRNA^VGlut^,Cas9.M6 | **0.136 ± 0.148 **** | -0.109 ± 0.116 | 0.284 ± 0.106 | -0.084 ± 0.061 | 61.9 ± 42.35 | 23.18 ± 0.27 | 4/24 |
| R18H11>Cas9.M6 | 0.25 ± 0.093 | -0.068 ± 0.124 | 0.387 ± 0.055 | -0.048 ± 0.057 | 55.46 ± 49.14 | 23.33 ± 0.43 | 5/20 |
| R51H05>sgRNA^VGlut^ | 0.348 ± 0.067 | -0.078 ± 0.078 | 0.398 ± 0.051 | -0.103 ± 0.047 | 122.02 ± 38.89 | 23.4 ± 0.37 | 0/20 |
| R51H05>sgRNA^VGlut^,Cas9.M6 | 0.26 ± 0.097 | -0.16 ± 0.116 | 0.312 ± 0.073 | -0.062 ± 0.059 | 82.56 ± 48.06 | 23.23 ± 0.33 | 2/22 |
| R51H05>Cas9.M6 | 0.251 ± 0.098 | -0.118 ± 0.143 | 0.349 ± 0.087 | -0.055 ± 0.072 | 45.76 ± 41.2 | 23.19 ± 0.39 | 3/17 |
| R79A11>sgRNA^VGlut^ | 0.172 ± 0.131 | -0.11 ± 0.102 | 0.378 ± 0.055 | -0.087 ± 0.062 | 88.87 ± 40.43 | 23.43 ± 0.31 | 1/21 |
| R79A11>sgRNA^VGlut^,Cas9.M6 | 0.178 ± 0.11 | -0.131 ± 0.083 | 0.325 ± 0.08 | -0.068 ± 0.037 | 75.67 ± 45.37 | 23.3 ± 0.29 | 1/19 |
| R79A11>Cas9.M6 | 0.19 ± 0.132 | -0.128 ± 0.114 | 0.346 ± 0.052 | -0.082 ± 0.065 | 31.29 ± 32.62 | 23.16 ± 0.47 | 3/21 |
| R91F02>sgRNA^VGlut^ | 0.233 ± 0.099 | -0.108 ± 0.1 | 0.341 ± 0.069 | -0.081 ± 0.049 | 97.13 ± 41.94 | 23.73 ± 0.39 | 0/21 |
| R91F02>sgRNA^VGlut^,Cas9.M6 | 0.301 ± 0.109 | -0.109 ± 0.056 | 0.381 ± 0.071 | -0.061 ± 0.042 | 112.28 ± 47.81 | 23.45 ± 0.31 | 0/24 |
| R91F02>Cas9.M6 | 0.26 ± 0.166 | -0.078 ± 0.107 | 0.366 ± 0.065 | -0.035 ± 0.04 | 72.82 ± 45.83 | 23.43 ± 0.41 | 2/22 |
| CNMa-KI-GAL4>sgRNA^VGlut^ | 0.28 ± 0.123 | -0.058 ± 0.088 | 0.357 ± 0.043 | -0.063 ± 0.019 | 54.7 ± 43.96 | 23.03 ± 0.33 | 3/23 |
| CNMa-KI-GAL4>sgRNA^VGlut^,Cas9.M6 | 0.268 ± 0.094 | -0.117 ± 0.119 | 0.331 ± 0.055 | -0.048 ± 0.086 | 51.21 ± 36.91 | 22.83 ± 0.29 | 3/18 |
| CNMa-KI-GAL4>Cas9.M6 | 0.261 ± 0.066 | -0.155 ± 0.114 | 0.342 ± 0.051 | -0.088 ± 0.08 | 54.14 ± 36.21 | 22.85 ± 0.21 | 2/24 |
| Clk4.1M>sgRNA^VGlut^ | 0.281 ± 0.116 | -0.052 ± 0.102 | 0.4 ± 0.057 | -0.174 ± 0.043 | 61.29 ± 46.5 | 23.45 ± 0.39 | 3/23 |
| Clk4.1M>sgRNA^VGlut^,Cas9.M6 | 0.181 ± 0.111 | -0.074 ± 0.07 | 0.313 ± 0.101 | -0.112 ± 0.047 | 77.13 ± 38.86 | 23.44 ± 0.38 | 0/21 |
| Clk4.1M>Cas9.M6 | 0.294 ± 0.102 | -0.096 ± 0.098 | 0.41 ± 0.035 | -0.087 ± 0.051 | 80.18 ± 40.76 | 23.51 ± 0.25 | 0/24 |

***** P＜0.01***
