# Supplementary material for: Conditional chemoconnectomics (cCCTomics) as a strategy for efficient and conditional targeting of chemical transmission
Source: eLife. 2024 Apr 30;12:RP91927. doi: 10.7554/eLife.91927 (PMC11060718; doi:10.7554/eLife.91927)
Supplement: Supplementary file 10. [file elife-91927-supp10.docx]

**Sequence of pBSK-attB-FRT-HpaI-T2A-EGFP-FRT, PM04, pMsgNull, Cas9.M0, Cas9.M6, Cas9.M9 and UAS-sgRNA structure**

pBSK-attB-FRT-HpaI-T2A-EGFP-FRT

**attB**-*FRT*-HpaI-T2A-***EGFP***-*FRT*

gcacttttcggggaaatgtgcgcggaacccctatttgtttatttttctaaatacattcaaatatgtatccgctcatgagacaataaccctgataaatgcttcaataatattgaaaaaggaagagtatgagtattcaacatttccgtgtcgcccttattcccttttttgcggcattttgccttcctgtttttgctcacccagaaacgctggtgaaagtaaaagatgctgaagatcagttgggtgcacgagtgggttacatcgaactggatctcaacagcggtaagatccttgagagttttcgccccgaagaacgttttccaatgatgagcacttttaaagttctgctatgtggcgcggtattatcccgtattgacgccgggcaagagcaactcggtcgccgcatacactattctcagaatgacttggttgagtactcaccagtcacagaaaagcatcttacggatggcatgacagtaagagaattatgcagtgctgccataaccatgagtgataacactgcggccaacttacttctgacaacgatcggaggaccgaaggagctaaccgcttttttgcacaacatgggggatcatgtaactcgccttgatcgttgggaaccggagctgaatgaagccataccaaacgacgagcgtgacaccacgatgcctgtagcaatggcaacaacgttgcgcaaactattaactggcgaactacttactctagcttcccggcaacaattaatagactggatggaggcggataaagttgcaggaccacttctgcgctcggcccttccggctggctggtttattgctgataaatctggagccggtgagcgtgggtctcgcggtatcattgcagcactggggccagatggtaagccctcccgtatcgtagttatctacacgacggggagtcaggcaactatggatgaacgaaatagacagatcgctgagataggtgcctcactgattaagcattggtaactgtcagaccaagtttactcatatatactttagattgatttaaaacttcatttttaatttaaaaggatctaggtgaagatcctttttgataatctcatgaccaaaatcccttaacgtgagttttcgttccactgagcgtcagaccccgtagaaaagatcaaaggatcttcttgagatcctttttttctgcgcgtaatctgctgcttgcaaacaaaaaaaccaccgctaccagcggtggtttgtttgccggatcaagagctaccaactctttttccgaaggtaactggcttcagcagagcgcagataccaaatactgtccttctagtgtagccgtagttaggccaccacttcaagaactctgtagcaccgcctacatacctcgctctgctaatcctgttaccagtggctgctgccagtggcgataagtcgtgtcttaccgggttggactcaagacgatagttaccggataaggcgcagcggtcgggctgaacggggggttcgtgcacacagcccagcttggagcgaacgacctacaccgaactgagatacctacagcgtgagctatgagaaagcgccacgcttcccgaagggagaaaggcggacaggtatccggtaagcggcagggtcggaacaggagagcgcacgagggagcttccagggggaaacgcctggtatctttatagtcctgtcgggtttcgccacctctgacttgagcgtcgatttttgtgatgctcgtcaggggggcggagcctatggaaaaacgccagcaacgcggcctttttacggttcctggccttttgctggccttttgctcacatgttctttcctgcgttatcccctgattctgtggataaccgtattaccgcctttgagtgagctgataccgctcgccgcagccgaacgaccgagcgcagcgagtcagtgagcgaggaagcggaagagcgcccaatacgcaaaccgcctctccccgcgcgttggccgattcattaatgcagctggcacgacaggtttcccgactggaaagcgggcagtgagcgcaacgcaattaatgtgagttagctcactcattaggcaccccaggctttacactttatgcttccggctcgtatgttgtgtggaattgtgagcggataacaatttcacacaggaaacagctatgaccatgattacgccaagctcgaaattaaccctcactaaagggaacaaaagctggagctcca**ccgcggtgcgggtgccagggcgtgcccttgggctccccgggcgcgtactccac***gaagttcctatactttctagagaataggaacttc*gttaacgcggccgcgggaagcggagaaggtcgtggtagtctactaacgtgtggtgacgtcgaggaaaatcctggacct***atggtgagcaagggcgaggagctgttcaccggggtggtgcccatcctggtcgagctggacggcgacgtaaacggccacaagttcagcgtgtccggcgagggcgagggcgatgccacctacggcaagctgaccctgaagttcatctgcaccaccggcaagctgcccgtgccctggcccaccctcgtgaccaccctgacctacggcgtgcagtgcttcagccgctaccccgaccacatgaagcagcacgacttcttcaagtccgccatgcccgaaggctacgtccaggagcgcaccatcttcttcaaggacgacggcaactacaagacccgcgccgaggtgaagttcgagggcgacaccctggtgaaccgcatcgagctgaagggcatcgacttcaaggaggacggcaacatcctggggcacaagctggagtacaactacaacagccacaacgtctatatcatggccgacaagcagaagaacggcatcaaggtgaacttcaagatccgccacaacatcgaggacggcagcgtgcagctcgccgaccactaccagcagaacacccccatcggcgacggccccgtgctgctgcccgacaaccactacctgagcacccagtccgccctgagcaaagaccccaacgagaagcgcgatcacatggtcctgctggagttcgtgaccgccgccgggatcactctcggcatggacgagctgtacaag***tagactagt*gaagttcctatactttctagagaataggaacttc*ggatcc**ataacttcgtatagcatacattatacgaagttat**cgtacgactagcacctgactgtcgagcccagtggaaacccttgaaatgcctttaagtcgagcccagtggaaacccttgaaatgcctttaagtcgagcccagtggaaacccttgaaatgcctttaagtcgagcccagtggaaacccttgaaatgcctttaagtcgagcccagtggaaacccttgaaatgcctttaagtcgagtagcagtcgaggtaggtacgtcatgccccgttattctctattcgttttgtgactctccctctctgtactattgctctctcactctgtcgcacagtaaacggcactctattctcgttgcttcgagagagcgcgcctcgaatgttcgcgaaaagagcgccggagtataaatagaggcgcttcgtcgacggagcgtcaattcaattcaaacaagcaaagtgaacacatcgcgaagcaagctgagcaaacaaacaagcgcagctgaacaagctaaacaatctgcaataaagtgcaagttaaagtgaatcaattaaaagtaaccaacaaccaagtaattaaactaaaaactgcaactactgaaatcaaccaagaagtcattattgaagacaagaagagaactctgaataggtcgatagcgtcaattcggcacgaggtttcgtgacgaagctccaagcggtttacgccatcaattaaacacaaagtgctgtgccaaaactcctctcgcttcttatttttgtttgttttttgagtgattgggtgggtgattggttttgggtgggtaagcaggggaaagtgtgaaaaatcccggcaatgggccaagaggatcaggagctattaattcgcggaggcagcaaacacccatctgccgagcatctgaacaatggtgacagcggagcggcttcgcagagctgcattaaccagggcttcgggcaggccaaaaactacggcacgctcctgccacccagtccgccggaggactccggttcagggagcggccaactagccgagaacctcacctatgcctggcacaatatggacatctttggggcggtcaatcagccgggctccggatggcggcagctggtcaaccggacacgcggactattctgcaacgagcgacacataccggcgcccaggaaacatttgctcaagaacgtttgcggcgtggcctatccgggcgaacttttggccgtgatgggcagttccggtgccggaaagacgaccctgctgaatgcccttgcctttcgatcgccgcagggcatccaagtatcgccatccgggatgcgactgctcaatggccaacctgtggacgccaaggagatgcaggccaggtgcgcctatgtccagcaggatgacctctttatcggctccctaacggccagggaacacctgattttccaagccatggtgcggatgccacgacatctgacctatcggcagcgagtggcccgcgtggatcaggtgatccaggagctttcgctcagcaaatgtcagcacacgatcatcggtgtgcccggcagggtgaaaggtctgtccggcggagaaaggaagcgtctggcattcgcctccgaggcactaaccgatccgccgcttctgatctgcgatgagcccacctccggactggactcatttaccgcccacagcgtcgtccaggtgctgaagaagctgtcgcagaagggcaagaccgtcatcctgaccattcatcagccgtcttccgagctgtttgagctctttgacaagatccttctgatggccgagggcagggtagctttcttgggcactcccagcgaagccgtcgacttcttttcctacgtgggtgcccagtgtcctaccaactacaatccggcggacttttacgtacaggtgttggccgttgtgcccggacgggagatcgagtcccgtgatcggatcgccaagatatgcgacaattttgccattagcaaagtagcccgggatatggagcagttgttggccaccaaaaatctggagaagccactggagcagccggagaatgggtacacctacaaggccacctggttcatgcagttccgggcggtcctgtggcgatcctggctgtcggtgctcaaggaaccactcctcgtaaaagtgcgacttattcagacaacgatggttgccatcttgattggcctcatctttttgggccaacaactcacgcaagtgggtgtgatgaatatcaacggagccatcttcctcttcctgaccaacatgacctttcaaaacgtctttgccacgataaatgtgttcacctcagagctgccagtttttatgagggaggcccgaagtcgactttatcgctgtgacacatactttctgggcaaaacgattgccgaattgccgctttttctcacagtgccactggtcttcacggcgattgcctatccgatgatcggactgcgggccggagtgctgcacttcttcaactgcctggcgctggtcactctggtggccaatgtgtcaacgtccttcggatatctaatatcctgcgccagctcctcgacctcgatggcgctgtctgtgggtccgccggttatcataccattcctgctctttggcggcttcttcttgaactcgggctcggtgccagtatacctcaaatggttgtcgtacctctcatggttccgttacgccaacgagggtctgctgattaaccaatgggcggacgtggagccgggcgaaattagctgcacatcgtcgaacaccacgtgccccagttcgggcaaggtcatcctggagacgcttaacttctccgccgccgatctgccgctggactacgtgggtctggccattctcatcgtgagcttccgggtgctcgcatatctggctctaagacttcgggcccgacgcaaggagtagccgacatatatccgaaataactgcttgttttttttttttaccattattaccatcgtgtttactgtttattgccccctcaaaaagctaatgtaattatatttgtgccaataaaaacaagatatgacctatagaatacaaaaaaaaaaaaaaaaaaaaaaaaaaaaactcgacaccgggatgaattcgatatcaagcttatcgataccgtcgacctcgagggggggcccggtacccaattcgccctatagtgagtcgtattacaattcactggccgtcgttttacaacgtcgtgactgggaaaaccctggcgttacccaacttaatcgccttgcagcacatccccctttcgccagctggcgtaatagcgaagaggcccgcaccgatcgcccttcccaacagttgcgcagcctgaatggcgaatgggacgcgccctgtagcggcgcattaagcgcggcgggtgtggtggttacgcgcagcgtgaccgctacacttgccagcgccctagcgcccgctcctttcgctttcttcccttcctttctcgccacgttcgccggctttccccgtcaagctctaaatcgggggctccctttagggttccgatttagtgctttacggcacctcgaccccaaaaaacttgattagggtgatggttcacgtagtgggccatcgccctgatagacggtttttcgccctttgacgttggagtccacgttctttaatagtggactcttgttccaaactggaacaacactcaaccctatctcggtctattcttttgatttataagggattttgccgatttcggcctattggttaaaaaatgagctgatttaacaaaaatttaacgcgaattttaacaaaatattaacgcttacaatttaggtg

PM04

ggccagacccacgtagtccagcggcagatcggcggcggagaagttaagcgtctccaggatgaccttgcccgaactggggcacgtggtgttcgacgatgtgcagctaatttcgcccggctccacgtccgcccattggttaatcagcagaccctcgttggcgtaacggaaccatgagaggtacgacaaccatttgaggtatactggcaccgagcccgagttcaagaagaaggcgtttttccataggctccgcccccctgacgagcatcacaaaaatcgacgctcaagtcagaggtggcgaaacccgacaggactataaagataccaggcgtttccccctggaagctccctcgtgcgctctcctgttccgaccctgccgcttaccggatacctgtccgcctttctcccttcgggaagcgtggcgctttctcaatgctcacgctgtaggtatctcagttcggtgtaggtcgttcgctccaagctgggctgtgtgcacgaaccccccgttcagcccgaccgctgcgccttatccggtaactatcgtcttgagtccaacccggtaagacacgacttatcgccactggcagcagccactggtaacaggattagcagagcgaggtatgtaggcggtgctacagagttcttgaagtggtggcctaactacggctacactagaaggacagtatttggtatctgcgctctgctgaagccagttaccttcggaaaaagagttggtagctcttgatccggcaaacaaaccaccgctggtagcggtggtttttttgtttgcaagcagcagattacgcgcagaaaaaaaggatctcaagaagatcctttgatcttttctacggggtctgacgctcagtggaacgaaaactcacgttaagggattttggtcatgagattatcaaaaaggatcttcacctagatccttttaaattaaaaatgaagttttaaatcaatctaaagtatatatgagtaaacttggtctgacagttaccaatgcttaatcagtgaggcacctatctcagcgatctgtctatttcgttcatccatagttgcctgactccccgtcgtgtagataactacgatacgggagggcttaccatctggccccagtgctgcaatgataccgcgagacccacgctcaccggctccagatttatcagcaataaaccagccagccggaagggccgagcgcagaagtggtcctgcaactttatccgcctccatccagtctattaattgttgccgggaagctagagtaagtagttcgccagttaatagtttgcgcaacgttgttgccattgctacaggcatcgtggtgtcacgctcgtcgtttggtatggcttcattcagctccggttcccaacgatcaaggcgagttacatgatcccccatgttgtgcaaaaaagcggttagctccttcggtcctccgatcgttgtcagaagtaagttggccgcagtgttatcactcatggttatggcagcactgcataattctcttactgtcatgccatccgtaagatgcttttctgtgactggtgagtactcaaccaagtcattctgagaatagtgtatgcggcgaccgagttgctcttgcccggcgtcaacacgggataataccgcgccacatagcagaactttaaaagtgctcatcattggaaaacgttcttcggggcgaaaactctcaaggatcttaccgctgttgagatccagttcgatgtaacccactcgtgcacccaactgatcttcagcatcttttactttcaccagcgtttctgggtgagcaaaaacaggaaggcaaaatgccgcaaaaaagggaataagggcgacacggaaatgttgaatactcatactcttcctttttcaatattattgaagcatttatcagggttattgtctcatgagcggatacatatttgaatgtatttagaaaaataaacaaataggggttccgcgcacatttccccgaaaagtgccacctgacgtctaagaaaccattattatcatgacattaacctataaaaataggcgtatcacgaggccctttcgtctcgcgcgtttcggtgatgacggtgaaaacctctgacacatgcagctcccggagacggtcacagcttgtctgtaagcggatgccgggagcagacaagcccgtcagggcgcgtcagcgggtgttggcgggtgtcggggctggcttaactatgcggcatcagagcagattgtactgagagtgcaccatatcgacatgcccgccgtgaccgtcgagaacccgctgacgctgccccgcgtatccgcacccgccgacgccgtcgcacgtcccgtgctcaccgtgaccaccgcgcccagcggtttcgagggcgagggcttcccggtgcgccgcgcgttcgccgggatcaactaccgccacctcgacccgttcatcatgatggaccagatgggtgaggtggagtacgcgcccggggagcccaagggcacgccctggcacccgcaccgcggcttcgagaccgtgacctacatcgtcgatatgcggtgtgaaataccgcaccgaatcgcgcggaactaacgacagtcgctccaaggtcgtcgaacaaaaggtgaatgtgttgcggagagcgggtgggagacagcgaaagagcaactacgaaacgtggtgtggtggaggtgaattatgaagagggcgcgcgatttgaaaagtatgtatataaaaaatatatcccggtgttttatgtagcgataaacgagtttttgatgtaaggtatgcaggtgtgtaagtcttttggttagaagacaaatccaaagtctacttgtggggatgttcgaaggggaaatacttgtattctataggtcatatcttgtttttattggcacaaatataattacattagctttttgagggggcaataaacagtaaacacgatggtaataatggtaaaaaaaaaaacaagcagttatttcggatatatgtcggctactccttgcgtcgggcccgaagtcttagagccagatatgcgagcacccggaagctcacgatgagaatggccagaccatgatgaaataacataaggtggtcccgtcggcaagagacatccacttaacgtatgcttgcaataagtgcgagtgaaaggaatagtattctgagtgtcgtattgagtctgagtgagacagcgatatgattgttgattaacccttagcatgtccgtggggtttgaattaactcataatattaattagacgaaattatttttaaagttttatttttaataatttgcgagtacgcaaagcttggctgcatccaacgcgttgggagctctccggatccaagcttgcatgcctgcaggtcggagtactgtcctccgagcggagtactgtcctccgagcggagtactgtcctccgagcggagtactgtcctccgagcggagtactgtcctccgagcggagactctagcgagcgccggagtataaatagaggcgcttcgtctacggagcgacaattcaattcaaacaagcaaagtgaacacgtcgctaagcgaaagctaagcaaataaacaagcgcagctgaacaagctaaacaatctgcagtaaagtgcaagttaaagtgaatcaattaaaagtaaccagcaaccaagtaaatcaactgcaactactgaaatctgccaagaagtaattattgaatacaagaagagaactctgaatagggaattgggaattgcagtgactctcttaaggtagccttgcagaagttggtcgtgaggcactgggcaggtaagtatcaaggttacaagacaggtttaaggagaccaatagaaactgggcttgtcgagacagagaagactcttgcgtttctgataggcacctattggtcttactgacatccactttgcctttctctccacaggtgtccactcccagttcaattacagctcgaattc**gtttaagagctatgctggaaacagcatagcaagtttaaataaggctagtccgttatcaacttgaaaaagtggcaccgagtcggtgcaacaaagcaccagtggtctagtggtagaatagtaccctgccacggtacagacccgggttcgattcccggctggtgca**ggtacctctagactaagccagtcggcaatcggacgccttcgaaacatgcaacactaatgtttaattcagatttcagcagagacaagctaaaacaccgacgagttgtaatcatttctgtgcgccagcatatatttcttatatacaacgtaatacataattatgtaattctagcatctccccaacactcacatacaaacaaacaaaaaatacaaacacacaaaacgtatttacccgcacgcatccttggcgaggttgagtatgaaacaaaaacaaaacttaatttagagcaaagtaattacacgaataaatttaataaaaaaaaactataataaaaacgccaatcatgttatttccaaaaaaaaaaaaaaaaaaaaaaactagacgagatgtttgcgcttgtgtcgatgaatgtaaagaattgcaactacatagatggtaattaatttcagctgtaagaagtacaagtttttcataattgtgagaaaagtgcagtagtatcgcttaaagttcagttttaatcagggcatttagcagctcgaacccaatgtatttgtaagcccaataaacgtgtaaatgcctagagcaaaatatcacaactgtagcttttcatccgaaagataataatgaatagactgacctgacgtttttcaaaatgcgtaggatccactagaaggccttagtatgtatgtaagttaataaaacccttttttggagaatgtagatttaaaaaaacatattttttttttattttttactgcactggacatcattgaacttatctgatcagttttaaatttacttcgatccaagggtatttgaagtaccaggttctttcgattacctctcactcaaaatgacattccactcaaagtcagcgctgtttgcctccttctctgtccacagaaatatcgccgtctctttcgccgctgcgtccgctatctctttcgccaccgtttgtagcgttacctagcgtcaatgtccgccttcagttgcactttgtcagcggtttcgtgacgaagctccaagcggtttacgccatcaattaaacacaaagtgctgtgccaaaactcctctcgcttcttatttttgtttgttttttgagtgattggggtggtgattggttttgggtgggtaagcaggggaaagtgtgaaaaatcccggcaatgggccaagaggatcaggagctattaattcgcggaggcagcaaacacccatctgccgagcatctgaacaatgtgagtagtacatgtgcatacatcttaagttcacttgatctataggaactgcgattgcaacatcaaattgtctgcggcgtgagaactgcgacccacaaaaatcccaaaccgcaatcgcacaaacaaatagtgacacgaaacagattattctggtagctgtgctcgctatataagacaatttttaagatcatatcatgatcaagacatctaaaggcattcattttcgactacattcttttttacaaaaaatataacaaccagatattttaagctgatcctagatgcacaaaaaataaataaaagtataaacctacttcgtaggatacttcgttttgttcggggttagatgagcataacgcttgtagttgatatttgagatcccctatcattgcagggtgacagcggagcggcttcgcagagctgcattaaccagggcttcgggcaggccaaaaactacggcacgctcctgccacccagtccgccggaggactccggttcagggagcggccaactagccgagaacctcacctatgcctggcacaatatggacatctttggggcggtcaatcagccgggctccggatggcggcagctggtcaaccggacacgcggactattctgcaacgagcgacacataccggcgcccaggaaacatttgctcaagaacggtgagtttctattcgcagtcggctgatctgtgtgaaatcttaataaagggtccaattaccaatttgaaactcagtttgcggcgtggcctatccgggcgaacttttggccgtgatgggcagttccggtgccggaaagacgaccctgctgaatgcccttgcctttcgatcgccgcagggcatccaagtatcgccatccgggatgcgactgctcaatggccaacctgtggacgccaaggagatgcaggccaggtgcgcctatgtccagcaggatgacctctttatcggctccctaacggccagggaacacctgattttccaggccatggtgcggatgccacgacatctgacctatcggcagcgagtggcccgcgtggatcaggtgatccaggagctttcgctcagcaaatgtcagcacacgatcatcggtgtgcccggcagggtgaaaggtctgtccggcggagaaaggaagcgtctggcattcgcctccgaggcactaaccgatccgccgcttctgatctgcgatgagcccacctccggactggactcatttaccgcccacagcgtcgtccaggtgctgaagaagctgtcgcagaagggcaagaccgtcatcctgaccattcatcagccgtcttccgagctgtttgagctctttgacaagatccttctgatggccgagggcagggtagctttcttgggcactcccagcgaagccgtcgacttcttttcctagtgagttcgatgtgtttattaagggtatctagcattacattacatctcaactcctatccagcgtgggtgcccagtgtcctaccaactacaatccggcggacttttacgtacaggtgttggccgttgtgcccggacgggagatcgagtcccgtgatcggatcgccaagatatgcgacaattttgctattagcaaagtagcccgggatatggagcagttgttggccaccaaaaatttggagaagccactggagcagccggagaatgggtacacctacaaggccacctggttcatgcagttccgggcggtcctgtggcgatcctggctgtcggtgctcaaggaaccactcctcgtaaaagtgcgacttattcagacaacggtgagtggttccagtggaaacaaatgatataacgcttacaattcttggaaacaaattcgctagattttagttagaattgcctgattccacacccttcttagtttttttcaatgagatgtatagtttatagttttgcagaaaataaataaatttcatttaactcgcgaacatgttgaagatatgaatattaatgagatgcgagtaacattttaatttgcagatggttgccatcttgattggcctcatctttttgggccaacaactcacgcaagtgggcgtgatgaatatcaacggagccatcttcctcttcctgaccaacatgacctttcaaaacgtctttgccacgataaatgtaagtcttgtttagaatacatttgcatattaataatttactaactttctaatgaatcgattcgatttaggtgttcacctcagagctgccagtttttatgagggaggcccgaagtcgactttatcgctgtgacacatactttctgggcaaaacgattgccgaattaccgctttttctcacagtgccactggtcttcacggcgattgcctatccgatgatcggactgcgggccggagtgctgcacttcttcaactgcctggcgctggtcactctggtggccaatgtgtcaacgtccttcggatatctaatatcctgcgccagctcctcgacctcgatggcgctgtctgtgggtccgccggttatcataccattcctgctctttggcggcttcttcttgaactcgggctcggtgccagtatacctcaaatggttgtcgtacctctcatggttccgttacgccaacgagggtctgctgattaaccaatgggcggacgtggagccgggcgaaattagctgcacatcgtcgaacaccacgtgccccagttcgggcaaggtcatcctggagacgcttaacttctccgccgccgatctgccgctggactacgtgggtctggccattctcatcgtgagcttccgggtgctcgcatatctggctctaagacttcgggcccgacgcaaggagtagccgacatatatccgaaataactgcttgtttttttttttaccattattaccatcgtgtttactgtttattgccccctcaaaaagctaatgtaattatatttgtgccaataaaaacaagatatgacctatagaatacaagtatttccccttcgaacatccccacaagtagactttggatttgtcttctaaccaaaagacttacacacctgcataccttacatcaaaaactcgtttatcgctacataaaacaccgggatatattttttatatacatacttttcaaatcgcgcgccctcttcataattcacctccaccacaccacgtttcgtagttgctctttcgctgtctcccacccgctctccgcaacacattcaccttttgttcgacgaccttggagcgactgtcgttagttccgcgcgattcggttcgctcaaatggttccgagtggttcatttcgtctcaatagaaattagtaataaatatttgtatgtacaatttatttgctccaatatatttgtatatatttccctcacagctatatttattctaatttaatattatgactttttaaggtaattttttgtgacctgttcggagtgattagcgttacaatttgaactgaaagtgacatccagtgtttgttccttgtgtagatgcatctcaaaaaaatggtgggcataatagtgttgtttatatatatcaaaaataacaactataataataagaatacatttaatttagaaaatgcttggatttcactggaactagaattaattcggctgctgctctaaacgacgcatttcgtactccaaagtacgaattttttccctcaagctcttattttcattaaacaatgaacaggacctaacgcacagtcacgttattgtttacataaatgattttttttactattcaaacttactctgtttgtgtactcccactggtatagccttcttttatcttttctggttcaggctctatcactttactaggtacggcatctgcgttgagtcgcctccttttaaatgtctgaccttttgcaggtgcagccttccactgcgaatcattaaagtgggtatcacaaatttgggagttttcaccaaggctgcacccaaggctctgctcccacaattttctcttaatagcacacttcggcacgtgaattaattttactccagtcacagctttgcagcaaaatttgcaatatttcatttttttttattccacgtaagggttaatgttttcaaaaaaaaattcgtccgcacacaacctttcctctcaacaagcaaacgtgcactgaatttaagtgtatacttcggtaagcttcggctatcgacgggaccaccttatgttatttcatcatg

pMsgNull

ggccagacccacgtagtccagcggcagatcggcggcggagaagttaagcgtctccaggatgaccttgcccgaactggggcacgtggtgttcgacgatgtgcagctaatttcgcccggctccacgtccgcccattggttaatcagcagaccctcgttggcgtaacggaaccatgagaggtacgacaaccatttgaggtatactggcaccgagcccgagttcaagaagaaggcgtttttccataggctccgcccccctgacgagcatcacaaaaatcgacgctcaagtcagaggtggcgaaacccgacaggactataaagataccaggcgtttccccctggaagctccctcgtgcgctctcctgttccgaccctgccgcttaccggatacctgtccgcctttctcccttcgggaagcgtggcgctttctcaatgctcacgctgtaggtatctcagttcggtgtaggtcgttcgctccaagctgggctgtgtgcacgaaccccccgttcagcccgaccgctgcgccttatccggtaactatcgtcttgagtccaacccggtaagacacgacttatcgccactggcagcagccactggtaacaggattagcagagcgaggtatgtaggcggtgctacagagttcttgaagtggtggcctaactacggctacactagaaggacagtatttggtatctgcgctctgctgaagccagttaccttcggaaaaagagttggtagctcttgatccggcaaacaaaccaccgctggtagcggtggtttttttgtttgcaagcagcagattacgcgcagaaaaaaaggatctcaagaagatcctttgatcttttctacggggtctgacgctcagtggaacgaaaactcacgttaagggattttggtcatgagattatcaaaaaggatcttcacctagatccttttaaattaaaaatgaagttttaaatcaatctaaagtatatatgagtaaacttggtctgacagttaccaatgcttaatcagtgaggcacctatctcagcgatctgtctatttcgttcatccatagttgcctgactccccgtcgtgtagataactacgatacgggagggcttaccatctggccccagtgctgcaatgataccgcgagacccacgctcaccggctccagatttatcagcaataaaccagccagccggaagggccgagcgcagaagtggtcctgcaactttatccgcctccatccagtctattaattgttgccgggaagctagagtaagtagttcgccagttaatagtttgcgcaacgttgttgccattgctacaggcatcgtggtgtcacgctcgtcgtttggtatggcttcattcagctccggttcccaacgatcaaggcgagttacatgatcccccatgttgtgcaaaaaagcggttagctccttcggtcctccgatcgttgtcagaagtaagttggccgcagtgttatcactcatggttatggcagcactgcataattctcttactgtcatgccatccgtaagatgcttttctgtgactggtgagtactcaaccaagtcattctgagaatagtgtatgcggcgaccgagttgctcttgcccggcgtcaacacgggataataccgcgccacatagcagaactttaaaagtgctcatcattggaaaacgttcttcggggcgaaaactctcaaggatcttaccgctgttgagatccagttcgatgtaacccactcgtgcacccaactgatcttcagcatcttttactttcaccagcgtttctgggtgagcaaaaacaggaaggcaaaatgccgcaaaaaagggaataagggcgacacggaaatgttgaatactcatactcttcctttttcaatattattgaagcatttatcagggttattgtctcatgagcggatacatatttgaatgtatttagaaaaataaacaaataggggttccgcgcacatttccccgaaaagtgccacctgacgtctaagaaaccattattatcatgacattaacctataaaaataggcgtatcacgaggccctttcgtctcgcgcgtttcggtgatgacggtgaaaacctctgacacatgcagctcccggagacggtcacagcttgtctgtaagcggatgccgggagcagacaagcccgtcagggcgcgtcagcgggtgttggcgggtgtcggggctggcttaactatgcggcatcagagcagattgtactgagagtgcaccatatcgacatgcccgccgtgaccgtcgagaacccgctgacgctgccccgcgtatccgcacccgccgacgccgtcgcacgtcccgtgctcaccgtgaccaccgcgcccagcggtttcgagggcgagggcttcccggtgcgccgcgcgttcgccgggatcaactaccgccacctcgacccgttcatcatgatggaccagatgggtgaggtggagtacgcgcccggggagcccaagggcacgccctggcacccgcaccgcggcttcgagaccgtgacctacatcgtcgatatgcggtgtgaaataccgcaccgaatcgcgcggaactaacgacagtcgctccaaggtcgtcgaacaaaaggtgaatgtgttgcggagagcgggtgggagacagcgaaagagcaactacgaaacgtggtgtggtggaggtgaattatgaagagggcgcgcgatttgaaaagtatgtatataaaaaatatatcccggtgttttatgtagcgataaacgagtttttgatgtaaggtatgcaggtgtgtaagtcttttggttagaagacaaatccaaagtctacttgtggggatgttcgaaggggaaatacttgtattctataggtcatatcttgtttttattggcacaaatataattacattagctttttgagggggcaataaacagtaaacacgatggtaataatggtaaaaaaaaaaacaagcagttatttcggatatatgtcggctactccttgcgtcgggcccgaagtcttagagccagatatgcgagcacccggaagctcacgatgagaatggccagaccatgatgaaataacataaggtggtcccgtcggcaagagacatccacttaacgtatgcttgcaataagtgcgagtgaaaggaatagtattctgagtgtcgtattgagtctgagtgagacagcgatatgattgttgattaacccttagcatgtccgtggggtttgaattaactcataatattaattagacgaaattatttttaaagttttatttttaataatttgcgagtacgcaaagcttggctgcatccaacgcgttgggagctctccggatccaagcttgcatgcctgcaggtcggagtactgtcctccgagcggagtactgtcctccgagcggagtactgtcctccgagcggagtactgtcctccgagcggagtactgtcctccgagcggagactctagcgagcgccggagtataaatagaggcgcttcgtctacggagcgacaattcaattcaaacaagcaaagtgaacacgtcgctaagcgaaagctaagcaaataaacaagcgcagctgaacaagctaaacaatctgcagtaaagtgcaagttaaagtgaatcaattaaaagtaaccagcaaccaagtaaatcaactgcaactactgaaatctgccaagaagtaattattgaatacaagaagagaactctgaatagggaattgggaattgcagtgactctcttaaggtagccttgcagaagttggtcgtgaggcactgggcaggtaagtatcaaggttacaagacaggtttaaggagaccaatagaaactgggcttgtcgagacagagaagactcttgcgtttctgataggcacctattggtcttactgacatccactttgcctttctctccacaggtgtccactcccagttcaattacagctcgaattcgggctttgagtgtgtgtagacatcaagcatcggtggttcagtggtagaatgctcgcctgccacgcgggcggcccgggttcgattcc***CGGCCG****gcagggccatcttaatgtcgcccttcagcacgccgtcctcggggtacaaccgctcggtggacgcttcccagcccattgtcttcttctgcattacggggccgtcaggagggaagttggtgccgcggagcttcaccttgtagatcagggtgccgtcctccagggaggtgtcctgggtcacggtcacggcgccgccgtcctcgaagttcatcacgcgctcccacttgaagccctcggggaaggactgcttatagtagtcggggatgtcggcggggtgcttggtgaaggccctggagccgtacatgaactgaggggacaggatgtcccaggagaagggcagggggccacccttggtcaccttcagcttggcggtctgggtgccctcgtaggggcggccctcgccctcgccctcgatctcgaactcgtggccgttcatggagccctccatgtgcaccttgaaccgcatgaactccttgatcactgcctcgcccttgctcaccatggtggctagcataacttcgtataaagtatcctatacgaagttatttgccttaacccagaaattatcactgttattctttagaatggtgcaaagaataacttcgtataatgtatgctatacgaagttatgaattcgatatcaagcttatcgataatcaacctctggattacaaaatttgtgaaagattgactggtattcttaactatgttgctccttttacgctatgtggatacgctgctttaatgcctttgtatcatgctattgcttcccgtatggctttcattttctcctccttgtataaatcctggttgctgtctctttatgaggagttgtggcccgttgtcaggcaacgtggcgtggtgtgcactgtgtttgctgacgcaacccccactggttggggcattgccaccacctgtcagctcctttccgggactttcgctttccccctccctattgccacggcggaactcatcgccgcctgccttgcccgctgctggacaggggctcggctgttgggcactgacaattccgtggtgttgtcggggaaatcatcgtcctttccttggctgctcgcctatgttgccacctggattctgcgcgggacgtccttctgctacgtcccttcggccctcaatccagcggaccttccttcccgcggcctgctgccggctctgcggcctcttccgcgtcttcgccttcgccctcagacgagtcggatctccctttgggccgcctccccgcatcgataccgagcgctgctcgagagatctacgggtggcatccctgtgacccctccccagtgcctctcctggccctggaagttgccactccagtgcccaccagccttgtcctaataaaattaagttgcatcattttgtctgactaggtgtccttctataatattatggggtggaggggggtggtatggagcaaggggcaagttgggaagacaacctgtagggcctgcggggtctattgggaaccaagctggagtgcagtggcacaatcttggctcactgcaatctccgcctcctgggttcaagcgattctcctgcctcagcctcccgagttgttgggattccaggcatgcatgaccaggctcagctaatttttgtttttttggtagagacggggtttcaccatattggccaggctggtctccaactcctaatctcaggtgatctacccaccttggcctcccaaattgctgggattacaggcgtgaaccactgctcccttccctgtcctt****GGTACC***tctagactaagccagtcggcaatcggacgccttcgaaacatgcaacactaatgtttaattcagatttcagcagagacaagctaaaacaccgacgagttgtaatcatttctgtgcgccagcatatatttcttatatacaacgtaatacataattatgtaattctagcatctccccaacactcacatacaaacaaacaaaaaatacaaacacacaaaacgtatttacccgcacgcatccttggcgaggttgagtatgaaacaaaaacaaaacttaatttagagcaaagtaattacacgaataaatttaataaaaaaaaactataataaaaacgccaatcatgttatttccaaaaaaaaaaaaaaaaaaaaaaactagacgagatgtttgcgcttgtgtcgatgaatgtaaagaattgcaactacatagatggtaattaatttcagctgtaagaagtacaagtttttcataattgtgagaaaagtgcagtagtatcgcttaaagttcagttttaatcagggcatttagcagctcgaacccaatgtatttgtaagcccaataaacgtgtaaatgcctagagcaaaatatcacaactgtagcttttcatccgaaagataataatgaatagactgacctgacgtttttcaaaatgcgtaggatccactagaaggccttagtatgtatgtaagttaataaaacccttttttggagaatgtagatttaaaaaaacatattttttttttattttttactgcactggacatcattgaacttatctgatcagttttaaatttacttcgatccaagggtatttgaagtaccaggttctttcgattacctctcactcaaaatgacattccactcaaagtcagcgctgtttgcctccttctctgtccacagaaatatcgccgtctctttcgccgctgcgtccgctatctctttcgccaccgtttgtagcgttacctagcgtcaatgtccgccttcagttgcactttgtcagcggtttcgtgacgaagctccaagcggtttacgccatcaattaaacacaaagtgctgtgccaaaactcctctcgcttcttatttttgtttgttttttgagtgattggggtggtgattggttttgggtgggtaagcaggggaaagtgtgaaaaatcccggcaatgggccaagaggatcaggagctattaattcgcggaggcagcaaacacccatctgccgagcatctgaacaatgtgagtagtacatgtgcatacatcttaagttcacttgatctataggaactgcgattgcaacatcaaattgtctgcggcgtgagaactgcgacccacaaaaatcccaaaccgcaatcgcacaaacaaatagtgacacgaaacagattattctggtagctgtgctcgctatataagacaatttttaagatcatatcatgatcaagacatctaaaggcattcattttcgactacattcttttttacaaaaaatataacaaccagatattttaagctgatcctagatgcacaaaaaataaataaaagtataaacctacttcgtaggatacttcgttttgttcggggttagatgagcataacgcttgtagttgatatttgagatcccctatcattgcagggtgacagcggagcggcttcgcagagctgcattaaccagggcttcgggcaggccaaaaactacggcacgctcctgccacccagtccgccggaggactccggttcagggagcggccaactagccgagaacctcacctatgcctggcacaatatggacatctttggggcggtcaatcagccgggctccggatggcggcagctggtcaaccggacacgcggactattctgcaacgagcgacacataccggcgcccaggaaacatttgctcaagaacggtgagtttctattcgcagtcggctgatctgtgtgaaatcttaataaagggtccaattaccaatttgaaactcagtttgcggcgtggcctatccgggcgaacttttggccgtgatgggcagttccggtgccggaaagacgaccctgctgaatgcccttgcctttcgatcgccgcagggcatccaagtatcgccatccgggatgcgactgctcaatggccaacctgtggacgccaaggagatgcaggccaggtgcgcctatgtccagcaggatgacctctttatcggctccctaacggccagggaacacctgattttccaggccatggtgcggatgccacgacatctgacctatcggcagcgagtggcccgcgtggatcaggtgatccaggagctttcgctcagcaaatgtcagcacacgatcatcggtgtgcccggcagggtgaaaggtctgtccggcggagaaaggaagcgtctggcattcgcctccgaggcactaaccgatccgccgcttctgatctgcgatgagcccacctccggactggactcatttaccgcccacagcgtcgtccaggtgctgaagaagctgtcgcagaagggcaagaccgtcatcctgaccattcatcagccgtcttccgagctgtttgagctctttgacaagatccttctgatggccgagggcagggtagctttcttgggcactcccagcgaagccgtcgacttcttttcctagtgagttcgatgtgtttattaagggtatctagcattacattacatctcaactcctatccagcgtgggtgcccagtgtcctaccaactacaatccggcggacttttacgtacaggtgttggccgttgtgcccggacgggagatcgagtcccgtgatcggatcgccaagatatgcgacaattttgctattagcaaagtagcccgggatatggagcagttgttggccaccaaaaatttggagaagccactggagcagccggagaatgggtacacctacaaggccacctggttcatgcagttccgggcggtcctgtggcgatcctggctgtcggtgctcaaggaaccactcctcgtaaaagtgcgacttattcagacaacggtgagtggttccagtggaaacaaatgatataacgcttacaattcttggaaacaaattcgctagattttagttagaattgcctgattccacacccttcttagtttttttcaatgagatgtatagtttatagttttgcagaaaataaataaatttcatttaactcgcgaacatgttgaagatatgaatattaatgagatgcgagtaacattttaatttgcagatggttgccatcttgattggcctcatctttttgggccaacaactcacgcaagtgggcgtgatgaatatcaacggagccatcttcctcttcctgaccaacatgacctttcaaaacgtctttgccacgataaatgtaagtcttgtttagaatacatttgcatattaataatttactaactttctaatgaatcgattcgatttaggtgttcacctcagagctgccagtttttatgagggaggcccgaagtcgactttatcgctgtgacacatactttctgggcaaaacgattgccgaattaccgctttttctcacagtgccactggtcttcacggcgattgcctatccgatgatcggactgcgggccggagtgctgcacttcttcaactgcctggcgctggtcactctggtggccaatgtgtcaacgtccttcggatatctaatatcctgcgccagctcctcgacctcgatggcgctgtctgtgggtccgccggttatcataccattcctgctctttggcggcttcttcttgaactcgggctcggtgccagtatacctcaaatggttgtcgtacctctcatggttccgttacgccaacgagggtctgctgattaaccaatgggcggacgtggagccgggcgaaattagctgcacatcgtcgaacaccacgtgccccagttcgggcaaggtcatcctggagacgcttaacttctccgccgccgatctgccgctggactacgtgggtctggccattctcatcgtgagcttccgggtgctcgcatatctggctctaagacttcgggcccgacgcaaggagtagccgacatatatccgaaataactgcttgtttttttttttaccattattaccatcgtgtttactgtttattgccccctcaaaaagctaatgtaattatatttgtgccaataaaaacaagatatgacctatagaatacaagtatttccccttcgaacatccccacaagtagactttggatttgtcttctaaccaaaagacttacacacctgcataccttacatcaaaaactcgtttatcgctacataaaacaccgggatatattttttatatacatacttttcaaatcgcgcgccctcttcataattcacctccaccacaccacgtttcgtagttgctctttcgctgtctcccacccgctctccgcaacacattcaccttttgttcgacgaccttggagcgactgtcgttagttccgcgcgattcggttcgctcaaatggttccgagtggttcatttcgtctcaatagaaattagtaataaatatttgtatgtacaatttatttgctccaatatatttgtatatatttccctcacagctatatttattctaatttaatattatgactttttaaggtaattttttgtgacctgttcggagtgattagcgttacaatttgaactgaaagtgacatccagtgtttgttccttgtgtagatgcatctcaaaaaaatggtgggcataatagtgttgtttatatatatcaaaaataacaactataataataagaatacatttaatttagaaaatgcttggatttcactggaactagaattaattcggctgctgctctaaacgacgcatttcgtactccaaagtacgaattttttccctcaagctcttattttcattaaacaatgaacaggacctaacgcacagtcacgttattgtttacataaatgattttttttactattcaaacttactctgtttgtgtactcccactggtatagccttcttttatcttttctggttcaggctctatcactttactaggtacggcatctgcgttgagtcgcctccttttaaatgtctgaccttttgcaggtgcagccttccactgcgaatcattaaagtgggtatcacaaatttgggagttttcaccaaggctgcacccaaggctctgctcccacaattttctcttaatagcacacttcggcacgtgaattaattttactccagtcacagctttgcagcaaaatttgcaatatttcatttttttttattccacgtaagggttaatgttttcaaaaaaaaattcgtccgcacacaacctttcctctcaacaagcaaacgtgcactgaatttaagtgtatacttcggtaagcttcggctatcgacgggaccaccttatgttatttcatcatg

Cas9.M0

Cas9-*GGSGP linker*-SV40NLS-**HA**

atggacaagaagtactccattgggctcgatatcggcacaaacagcgtcggctgggccgtcattacggacgagtacaaggtgccgagcaaaaaattcaaagttctgggcaataccgatcgccacagcataaagaagaacctcattggcgccctcctgttcgactccggggagacggccgaagccacgcggctcaaaagaacagcacggcgcagatatacccgcagaaagaatcggatctgctacctgcaggagatctttagtaatgagatggctaaggtggatgactctttcttccataggctggaggagtcctttttggtggaggaggataaaaagcacgagcgccacccaatctttggcaatatcgtggacgaggtggcgtaccatgaaaagtacccaaccatatatcatctgaggaagaagcttgtagacagtactgataaggctgacttgcggttgatctatctcgcgctggcgcatatgatcaaatttcggggacacttcctcatcgagggggacctgaacccagacaacagcgatgtcgacaaactctttatccaactggttcagacttacaatcagcttttcgaagagaacccgatcaacgcatccggagttgacgccaaagcaatcctgagcgctaggctgtccaaatcccggcggctcgaaaacctcatcgcacagctccctggggagaagaagaacggcctgtttggtaatcttatcgccctgtcactcgggctgacccccaactttaaatctaacttcgacctggccgaagatgccaagcttcaactgagcaaagacacctacgatgatgatctcgacaatctgctggcccagatcggcgaccagtacgcagacctttttttggcggcaaagaacctgtcagacgccattctgctgagtgatattctgcgagtgaacacggagatcaccaaagctccgctgagcgctagtatgatcaagcgctatgatgagcaccaccaagacttgactttgctgaaggcccttgtcagacagcaactgcctgagaagtacaaggaaattttcttcgatcagtctaaaaatggctacgccggatacattgacggcggagcaagccaggaggaattttacaaatttattaagcccatcttggaaaaaatggacggcaccgaggagctgctggtaaagcttaacagagaagatctgttgcgcaaacagcgcactttcgacaatggaagcatcccccaccagattcacctgggcgaactgcacgctatcctcaggcggcaagaggatttctacccctttttgaaagataacagggaaaagattgagaaaatcctcacatttcggataccctactatgtaggccccctcgcccggggaaattccagattcgcgtggatgactcgcaaatcagaagagaccatcactccctggaacttcgaggaagtcgtggataagggggcctctgcccagtccttcatcgaaaggatgactaactttgataaaaatctgcctaacgaaaaggtgcttcctaaacactctctgctgtacgagtacttcacagtttataacgagctcaccaaggtcaaatacgtcacagaagggatgagaaagccagcattcctgtctggagagcagaagaaagctatcgtggacctcctcttcaagacgaaccggaaagttaccgtgaaacagctcaaagaagactatttcaaaaagattgaatgtttcgactctgttgaaatcagcggagtggaggatcgcttcaacgcatccctgggaacgtatcacgatctcctgaaaatcattaaagacaaggacttcctggacaatgaggagaacgaggacattcttgaggacattgtcctcacccttacgttgtttgaagatagggagatgattgaagaacgcttgaaaacttacgctcatctcttcgacgacaaagtcatgaaacagctcaagaggcgccgatatacaggatgggggcggctgtcaagaaaactgatcaatgggatccgagacaagcagagtggaaagacaatcctggattttcttaagtccgatggatttgccaaccggaacttcatgcagttgatccatgatgactctctcacctttaaggaggacatccagaaagcacaagtttctggccagggggacagtcttcacgagcacatcgctaatcttgcaggtagcccagctatcaaaaagggaatactgcagaccgttaaggtcgtggatgaactcgtcaaagtaatgggaaggcataagcccgagaatatcgttatcgagatggcccgagagaaccaaactacccagaagggacagaagaacagtagggaaaggatgaagaggattgaagagggtataaaagaactggggtcccaaatccttaaggaacacccagttgaaaacacccagcttcagaatgagaagctctacctgtactacctgcagaacggcagggacatgtacgtggatcaggaactggacatcaatcggctctccgactacgacgtggatcatatcgtgccccagtcttttctcaaagatgattctattgataataaagtgttgacaagatccgataaaaatagagggaagagtgataacgtcccctcagaagaagttgtcaagaaaatgaaaaattattggcggcagctgctgaacgccaaactgatcacacaacggaagttcgataatctgactaaggctgaacgaggtggcctgtctgagttggataaagccggcttcatcaaaaggcagcttgttgagacacgccagatcaccaagcacgtggcccaaattctcgattcacgcatgaacaccaagtacgatgaaaatgacaaactgattcgagaggtgaaagttattactctgaagtctaagctggtctcagatttcagaaaggactttcagttttataaggtgagagagatcaacaattaccaccatgcgcatgatgcctacctgaatgcagtggtaggcactgcacttatcaaaaaatatcccaagcttgaatctgaatttgtttacggagactataaagtgtacgatgttaggaaaatgatcgcaaagtctgagcaggaaataggcaaggccaccgctaagtacttcttttacagcaatattatgaattttttcaagaccgagattacactggccaatggagagattcggaagcgaccacttatcgaaacaaacggagaaacaggagaaatcgtgtgggacaagggtagggatttcgcgacagtccggaaggtcctgtccatgccgcaggtgaacatcgttaaaaagaccgaagtacagaccggaggcttctccaaggaaagtatcctcccgaaaaggaacagcgacaagctgatcgcacgcaaaaaagattgggaccccaagaaatacggcggattcgattctcctacagtcgcttacagtgtactggttgtggccaaagtggagaaagggaagtctaaaaaactcaaaagcgtcaaggaactgctgggcatcacaatcatggagcgatcaagcttcgaaaaaaaccccatcgactttctcgaggcgaaaggatataaagaggtcaaaaaagacctcatcattaagcttcccaagtactctctctttgagcttgaaaacggccggaaacgaatgctcgctagtgcgggcgagctgcagaaaggtaacgagctggcactgccctctaaatacgttaatttcttgtatctggccagccactatgaaaagctcaaagggtctcccgaagataatgagcagaagcagctgttcgtggaacaacacaaacactaccttgatgagatcatcgagcaaataagcgaattctccaaaagagtgatcctcgccgacgctaacctcgataaggtgctttctgcttacaataagcacagggataagcccatcagggagcaggcagaaaacattatccacttgtttactctgaccaacttgggcgcgcctgcagccttcaagtacttcgacaccaccatagacagaaagcggtacacctctacaaaggaggtcctggacgccacactgattcatcagtcaattacggggctctatgaaacaagaatcgacctctctcagctcggtggagac*ggcggttcgggtccg*cccaagaagaaacgtaaggtt**tatccctatgatgtgccggattatgcg**tga

Cas9.M6

HMGN1 region-*GGSGP linker*-Cas9- *GGSGP linker***-UDP(undefined peptide) region**

atgcccaagaggaaggtcagctccgccgaaggcgccgccaaggaagagcccaagaggagatcggcgcggttgtcagctaaacctcctgcaaaagtggaagcgaagccgaaaaaggcagcagcgaaggataaatcttcagacaaaaaagtgcaaacaaaagggaaaaggggagcaaagggaaaacaggccgaagtggctaaccaagaaactaaagaagacttacctgcggaaaacggggaaacgaagactgaggagagtccagcctctgatgaagcaggagagaaagaagccaagtctgat*ggcggttcgggtccg*atggacaagaagtactccattgggctcgatatcggcacaaacagcgtcggctgggccgtcattacggacgagtacaaggtgccgagcaaaaaattcaaagttctgggcaataccgatcgccacagcataaagaagaacctcattggcgccctcctgttcgactccggggagacggccgaagccacgcggctcaaaagaacagcacggcgcagatatacccgcagaaagaatcggatctgctacctgcaggagatctttagtaatgagatggctaaggtggatgactctttcttccataggctggaggagtcctttttggtggaggaggataaaaagcacgagcgccacccaatctttggcaatatcgtggacgaggtggcgtaccatgaaaagtacccaaccatatatcatctgaggaagaagcttgtagacagtactgataaggctgacttgcggttgatctatctcgcgctggcgcatatgatcaaatttcggggacacttcctcatcgagggggacctgaacccagacaacagcgatgtcgacaaactctttatccaactggttcagacttacaatcagcttttcgaagagaacccgatcaacgcatccggagttgacgccaaagcaatcctgagcgctaggctgtccaaatcccggcggctcgaaaacctcatcgcacagctccctggggagaagaagaacggcctgtttggtaatcttatcgccctgtcactcgggctgacccccaactttaaatctaacttcgacctggccgaagatgccaagcttcaactgagcaaagacacctacgatgatgatctcgacaatctgctggcccagatcggcgaccagtacgcagacctttttttggcggcaaagaacctgtcagacgccattctgctgagtgatattctgcgagtgaacacggagatcaccaaagctccgctgagcgctagtatgatcaagcgctatgatgagcaccaccaagacttgactttgctgaaggcccttgtcagacagcaactgcctgagaagtacaaggaaattttcttcgatcagtctaaaaatggctacgccggatacattgacggcggagcaagccaggaggaattttacaaatttattaagcccatcttggaaaaaatggacggcaccgaggagctgctggtaaagcttaacagagaagatctgttgcgcaaacagcgcactttcgacaatggaagcatcccccaccagattcacctgggcgaactgcacgctatcctcaggcggcaagaggatttctacccctttttgaaagataacagggaaaagattgagaaaatcctcacatttcggataccctactatgtaggccccctcgcccggggaaattccagattcgcgtggatgactcgcaaatcagaagagaccatcactccctggaacttcgaggaagtcgtggataagggggcctctgcccagtccttcatcgaaaggatgactaactttgataaaaatctgcctaacgaaaaggtgcttcctaaacactctctgctgtacgagtacttcacagtttataacgagctcaccaaggtcaaatacgtcacagaagggatgagaaagccagcattcctgtctggagagcagaagaaagctatcgtggacctcctcttcaagacgaaccggaaagttaccgtgaaacagctcaaagaagactatttcaaaaagattgaatgtttcgactctgttgaaatcagcggagtggaggatcgcttcaacgcatccctgggaacgtatcacgatctcctgaaaatcattaaagacaaggacttcctggacaatgaggagaacgaggacattcttgaggacattgtcctcacccttacgttgtttgaagatagggagatgattgaagaacgcttgaaaacttacgctcatctcttcgacgacaaagtcatgaaacagctcaagaggcgccgatatacaggatgggggcggctgtcaagaaaactgatcaatgggatccgagacaagcagagtggaaagacaatcctggattttcttaagtccgatggatttgccaaccggaacttcatgcagttgatccatgatgactctctcacctttaaggaggacatccagaaagcacaagtttctggccagggggacagtcttcacgagcacatcgctaatcttgcaggtagcccagctatcaaaaagggaatactgcagaccgttaaggtcgtggatgaactcgtcaaagtaatgggaaggcataagcccgagaatatcgttatcgagatggcccgagagaaccaaactacccagaagggacagaagaacagtagggaaaggatgaagaggattgaagagggtataaaagaactggggtcccaaatccttaaggaacacccagttgaaaacacccagcttcagaatgagaagctctacctgtactacctgcagaacggcagggacatgtacgtggatcaggaactggacatcaatcggctctccgactacgacgtggatcatatcgtgccccagtcttttctcaaagatgattctattgataataaagtgttgacaagatccgataaaaatagagggaagagtgataacgtcccctcagaagaagttgtcaagaaaatgaaaaattattggcggcagctgctgaacgccaaactgatcacacaacggaagttcgataatctgactaaggctgaacgaggtggcctgtctgagttggataaagccggcttcatcaaaaggcagcttgttgagacacgccagatcaccaagcacgtggcccaaattctcgattcacgcatgaacaccaagtacgatgaaaatgacaaactgattcgagaggtgaaagttattactctgaagtctaagctggtctcagatttcagaaaggactttcagttttataaggtgagagagatcaacaattaccaccatgcgcatgatgcctacctgaatgcagtggtaggcactgcacttatcaaaaaatatcccaagcttgaatctgaatttgtttacggagactataaagtgtacgatgttaggaaaatgatcgcaaagtctgagcaggaaataggcaaggccaccgctaagtacttcttttacagcaatattatgaattttttcaagaccgagattacactggccaatggagagattcggaagcgaccacttatcgaaacaaacggagaaacaggagaaatcgtgtgggacaagggtagggatttcgcgacagtccggaaggtcctgtccatgccgcaggtgaacatcgttaaaaagaccgaagtacagaccggaggcttctccaaggaaagtatcctcccgaaaaggaacagcgacaagctgatcgcacgcaaaaaagattgggaccccaagaaatacggcggattcgattctcctacagtcgcttacagtgtactggttgtggccaaagtggagaaagggaagtctaaaaaactcaaaagcgtcaaggaactgctgggcatcacaatcatggagcgatcaagcttcgaaaaaaaccccatcgactttctcgaggcgaaaggatataaagaggtcaaaaaagacctcatcattaagcttcccaagtactctctctttgagcttgaaaacggccggaaacgaatgctcgctagtgcgggcgagctgcagaaaggtaacgagctggcactgccctctaaatacgttaatttcttgtatctggccagccactatgaaaagctcaaagggtctcccgaagataatgagcagaagcagctgttcgtggaacaacacaaacactaccttgatgagatcatcgagcaaataagcgaattctccaaaagagtgatcctcgccgacgctaacctcgataaggtgctttctgcttacaataagcacagggataagcccatcagggagcaggcagaaaacattatccacttgtttactctgaccaacttgggcgcgcctgcagccttcaagtacttcgacaccaccatagacagaaagcggtacacctctacaaaggaggtcctggacgccacactgattcatcagtcaattacggggctctatgaaacaagaatcgacctctctcagctcggtggagac*ggcggttcgggtccg***TccaCAGACCACCCCAAGTCTCTCAGCTCGGTGGAGACGGCGGTTCGGGTCCGTCCACAGACCACCCCAAGTCTCTCAGCTCGGTGGAGACGGCGGTTCGGGTCCGTCCAAGAAGAAGAGGAAGGTGTGAGGTACCTC**tag

Cas9.M9

HMGN1 region-*GGSGP linker*-Cas9- *GGSGP linker***-HMGB1 region-***GGSGP linker-*SV40NLS

atgcccaagaggaaggtcagctccgccgaaggcgccgccaaggaagagcccaagaggagatcggcgcggttgtcagctaaacctcctgcaaaagtggaagcgaagccgaaaaaggcagcagcgaaggataaatcttcagacaaaaaagtgcaaacaaaagggaaaaggggagcaaagggaaaacaggccgaagtggctaaccaagaaactaaagaagacttacctgcggaaaacggggaaacgaagactgaggagagtccagcctctgatgaagcaggagagaaagaagccaagtctgat*ggcggttcgggtccg*atggacaagaagtactccattgggctcgatatcggcacaaacagcgtcggctgggccgtcattacggacgagtacaaggtgccgagcaaaaaattcaaagttctgggcaataccgatcgccacagcataaagaagaacctcattggcgccctcctgttcgactccggggagacggccgaagccacgcggctcaaaagaacagcacggcgcagatatacccgcagaaagaatcggatctgctacctgcaggagatctttagtaatgagatggctaaggtggatgactctttcttccataggctggaggagtcctttttggtggaggaggataaaaagcacgagcgccacccaatctttggcaatatcgtggacgaggtggcgtaccatgaaaagtacccaaccatatatcatctgaggaagaagcttgtagacagtactgataaggctgacttgcggttgatctatctcgcgctggcgcatatgatcaaatttcggggacacttcctcatcgagggggacctgaacccagacaacagcgatgtcgacaaactctttatccaactggttcagacttacaatcagcttttcgaagagaacccgatcaacgcatccggagttgacgccaaagcaatcctgagcgctaggctgtccaaatcccggcggctcgaaaacctcatcgcacagctccctggggagaagaagaacggcctgtttggtaatcttatcgccctgtcactcgggctgacccccaactttaaatctaacttcgacctggccgaagatgccaagcttcaactgagcaaagacacctacgatgatgatctcgacaatctgctggcccagatcggcgaccagtacgcagacctttttttggcggcaaagaacctgtcagacgccattctgctgagtgatattctgcgagtgaacacggagatcaccaaagctccgctgagcgctagtatgatcaagcgctatgatgagcaccaccaagacttgactttgctgaaggcccttgtcagacagcaactgcctgagaagtacaaggaaattttcttcgatcagtctaaaaatggctacgccggatacattgacggcggagcaagccaggaggaattttacaaatttattaagcccatcttggaaaaaatggacggcaccgaggagctgctggtaaagcttaacagagaagatctgttgcgcaaacagcgcactttcgacaatggaagcatcccccaccagattcacctgggcgaactgcacgctatcctcaggcggcaagaggatttctacccctttttgaaagataacagggaaaagattgagaaaatcctcacatttcggataccctactatgtaggccccctcgcccggggaaattccagattcgcgtggatgactcgcaaatcagaagagaccatcactccctggaacttcgaggaagtcgtggataagggggcctctgcccagtccttcatcgaaaggatgactaactttgataaaaatctgcctaacgaaaaggtgcttcctaaacactctctgctgtacgagtacttcacagtttataacgagctcaccaaggtcaaatacgtcacagaagggatgagaaagccagcattcctgtctggagagcagaagaaagctatcgtggacctcctcttcaagacgaaccggaaagttaccgtgaaacagctcaaagaagactatttcaaaaagattgaatgtttcgactctgttgaaatcagcggagtggaggatcgcttcaacgcatccctgggaacgtatcacgatctcctgaaaatcattaaagacaaggacttcctggacaatgaggagaacgaggacattcttgaggacattgtcctcacccttacgttgtttgaagatagggagatgattgaagaacgcttgaaaacttacgctcatctcttcgacgacaaagtcatgaaacagctcaagaggcgccgatatacaggatgggggcggctgtcaagaaaactgatcaatgggatccgagacaagcagagtggaaagacaatcctggattttcttaagtccgatggatttgccaaccggaacttcatgcagttgatccatgatgactctctcacctttaaggaggacatccagaaagcacaagtttctggccagggggacagtcttcacgagcacatcgctaatcttgcaggtagcccagctatcaaaaagggaatactgcagaccgttaaggtcgtggatgaactcgtcaaagtaatgggaaggcataagcccgagaatatcgttatcgagatggcccgagagaaccaaactacccagaagggacagaagaacagtagggaaaggatgaagaggattgaagagggtataaaagaactggggtcccaaatccttaaggaacacccagttgaaaacacccagcttcagaatgagaagctctacctgtactacctgcagaacggcagggacatgtacgtggatcaggaactggacatcaatcggctctccgactacgacgtggatcatatcgtgccccagtcttttctcaaagatgattctattgataataaagtgttgacaagatccgataaaaatagagggaagagtgataacgtcccctcagaagaagttgtcaagaaaatgaaaaattattggcggcagctgctgaacgccaaactgatcacacaacggaagttcgataatctgactaaggctgaacgaggtggcctgtctgagttggataaagccggcttcatcaaaaggcagcttgttgagacacgccagatcaccaagcacgtggcccaaattctcgattcacgcatgaacaccaagtacgatgaaaatgacaaactgattcgagaggtgaaagttattactctgaagtctaagctggtctcagatttcagaaaggactttcagttttataaggtgagagagatcaacaattaccaccatgcgcatgatgcctacctgaatgcagtggtaggcactgcacttatcaaaaaatatcccaagcttgaatctgaatttgtttacggagactataaagtgtacgatgttaggaaaatgatcgcaaagtctgagcaggaaataggcaaggccaccgctaagtacttcttttacagcaatattatgaattttttcaagaccgagattacactggccaatggagagattcggaagcgaccacttatcgaaacaaacggagaaacaggagaaatcgtgtgggacaagggtagggatttcgcgacagtccggaaggtcctgtccatgccgcaggtgaacatcgttaaaaagaccgaagtacagaccggaggcttctccaaggaaagtatcctcccgaaaaggaacagcgacaagctgatcgcacgcaaaaaagattgggaccccaagaaatacggcggattcgattctcctacagtcgcttacagtgtactggttgtggccaaagtggagaaagggaagtctaaaaaactcaaaagcgtcaaggaactgctgggcatcacaatcatggagcgatcaagcttcgaaaaaaaccccatcgactttctcgaggcgaaaggatataaagaggtcaaaaaagacctcatcattaagcttcccaagtactctctctttgagcttgaaaacggccggaaacgaatgctcgctagtgcgggcgagctgcagaaaggtaacgagctggcactgccctctaaatacgttaatttcttgtatctggccagccactatgaaaagctcaaagggtctcccgaagataatgagcagaagcagctgttcgtggaacaacacaaacactaccttgatgagatcatcgagcaaataagcgaattctccaaaagagtgatcctcgccgacgctaacctcgataaggtgctttctgcttacaataagcacagggataagcccatcagggagcaggcagaaaacattatccacttgtttactctgaccaacttgggcgcgcctgcagccttcaagtacttcgacaccaccatagacagaaagcggtacacctctacaaaggaggtcctggacgccacactgattcatcagtcaattacggggctctatgaaacaagaatcgacctctctcagctcggtggagac*ggcggttcgggtccg***atgggcaaaggagatcctaagaagccgagaggcaaaatgtcatcatatgcattttttgtgcaaacttgtcgggaggagcataagaagaagcacccagatgcttcagtcaacttctcagagttttctaagaagtgctcagagaggtggaagaccatgtctgctaaagagaaaggaaaatttgaagatatggcaaaagcggacaaggcccgttatgaaagagaaatgaaaacctatatccctcccaaaggggag***ggcggttcgggtccg*cccaagaagaagaggaaggtgtga

**UAS-sgRNA structure**

**tRNA**-sgRNA1-*tRNA*-sgRNA2-*tRNA*-sgRNA3-*tRNA*

**gggctttgagtgtgtgtagacatcaagcatcggtggttcagtggtagaatgctcgcctgccacgcgggcggcccgggttcgattcccggccgatgca**-N_20_-gtttaagagctatgctggaaacagcatagcaagtttaaataaggctagtccgttatcaacttgaaaaagtggcaccgagtcggtgc*aacaaagcaccagtggtctagtggtagaatagtaccctgccacggtacagacccgggttcgattcccggctggtgca*-N_20_-gtttaagagctatgctggaaacagcatagcaagtttaaataaggctagtccgttatcaacttgaaaaagtggcaccgagtcggtgc*aacaaagcaccagtggtctagtggtagaatagtaccctgccacggtacagacccgggttcgattcccggctggtgca*-N20-gtttaagagctatgctggaaacagcatagcaagtttaaataaggctagtccgttatcaacttgaaaaagtggcaccgagtcggtgc*aacaaagcaccagtggtctagtggtagaatagtaccctgccacggtacagacccgggttcgattcccggctggtgca*
